# BATF-dependent Th17 cells act through the IL-23R pathway to promote prostate adenocarcinoma initiation and progression

**DOI:** 10.1093/jnci/djae120

**Published:** 2024-06-04

**Authors:** Sen Liu, Seleste L Rivero, Bing Zhang, Keyi Shen, Zixuan Li, Tianhua Niu, Brian G Rowan, S Michal Jazwinski, Asim B Abdel-Mageed, Chad Steele, Alun R Wang, Oliver Sartor, Qiuyang Zhang

**Affiliations:** Department of Structural & Cellular Biology, Tulane University School of Medicine, New Orleans, LA, USA; Department of Structural & Cellular Biology, Tulane University School of Medicine, New Orleans, LA, USA; Department of Structural & Cellular Biology, Tulane University School of Medicine, New Orleans, LA, USA; Medical Laboratory of ShenZhen LuoHu People’s Hospital, The Third Affiliated Hospital of Shenzhen University, Shenzhen, China; Department of Structural & Cellular Biology, Tulane University School of Medicine, New Orleans, LA, USA; Department of Structural & Cellular Biology, Tulane University School of Medicine, New Orleans, LA, USA; Hubei University of Medicine, Shiyan, Hubei, China; Department of Biochemistry and Molecular Biology, Tulane University School of Medicine, New Orleans, LA, USA; Department of Structural & Cellular Biology, Tulane University School of Medicine, New Orleans, LA, USA; John W. Deming Department of Medicine, Tulane University School of Medicine, New Orleans, LA, USA; Tulane Center for Aging, Tulane University, New Orleans, LA, USA; Department of Urology, Tulane University School of Medicine, New Orleans, LA, USA; Department of Microbiology & Immunology, Tulane University School of Medicine, New Orleans, LA, USA; Department of Pathology and Laboratory Medicine, Tulane University School of Medicine, New Orleans, LA, USA; Department of Urology, Tulane University School of Medicine, New Orleans, LA, USA; Department of Medical Oncology, Mayo Clinic, Rochester, MN, USA; Department of Structural & Cellular Biology, Tulane University School of Medicine, New Orleans, LA, USA; Tulane Center for Aging, Tulane University, New Orleans, LA, USA; Tulane Cancer Center and Louisiana Cancer Research Center, Tulane University, New Orleans, LA, USA

## Abstract

**Background:**

The role of Th17 cells in prostate cancer is not fully understood. The transcription factor BATF controls the differentiation of Th17 cells. Mice deficient in Batf do not produce Th17 cells.

**Methods:**

In this study, we aimed to characterize the role of *Batf*-dependent Th17 cells in prostate cancer by crossbreeding *Batf* knockout mice with mice conditionally mutant for *Pten.*

**Results:**

We found that *Batf* knockout mice had changes in the morphology of prostate epithelial cells compared with normal mice, and *Batf* knockout mice deficient in *Pten* (called *Batf-*) had smaller prostate size and developed fewer invasive prostate adenocarcinomas than *Pten*-deficient mice with *Batf* expression (called *Batf+*). The prostate tumors in *Batf-* mice showed reduced proliferation, increased apoptosis, decreased angiogenesis and inflammatory cell infiltration, and activation of nuclear factor–κB signaling. Moreover, *Batf-* mice showed significantly reduced interleukin 23 (IL-23)-IL-23R signaling. In the prostate stroma of *Batf-* mice, IL-23R–positive cells were decreased considerably compared with *Batf*+ mice. Splenocytes and prostate tissues from *Batf-* mice cultured under Th17 differentiation conditions expressed reduced IL-23/IL-23R than cultured cells from *Batf*+ mice. Anti–IL-23p19 antibody treatment of *Pten*-deficient mice reduced prostate tumors and angiogenesis compared with control immunoglobulin G–treated mice. In human prostate tumors, BATF messenger RNA level was positively correlated with IL-23A and IL-23R but not RORC.

**Conclusion:**

Our novel findings underscore the crucial role of IL-23-IL-23R signaling in mediating the function of *Batf*-dependent Th17 cells, thereby promoting prostate cancer initiation and progression. This finding highlights the BATF–IL-23R axis as a promising target for the development of innovative strategies for prostate cancer prevention and treatment.

Prostate cancer remains the second-most common cancer and the fifth-leading cause of cancer death among men worldwide (1). Inflammation is frequently observed in clinical prostate specimens (2,3), but the mechanisms of how inflammation modulates prostate cancer remain unclear. Th17 cells are critical mediators of autoimmune and proinflammatory diseases (4). Previous studies indicated that Th17 cells increase in prostate cancer (5), and a higher percentage of Th17 cells in blood is correlated with poorer outcomes (6). Still, the role of Th17 in prostate cancer remains controversial (7). We have shown that interleukin 17 (IL-17) promotes the formation and growth of prostate adenocarcinoma in animal models (8), but IL-17 can be produced by many immune cells except CD4^+^ Th17 cells, including CD8^+^ T cells, γθ T cells, iNKT cells, ILC3, neutrophils, and mast cells (9). Therefore, the role of IL-17 may not represent the role of Th17 cells in cancer.

BATF, a member of the activator protein 1 family of transcription factors, is predominantly expressed in cells of hematopoietic origin, especially in multiple helper T-cell subsets, and cooperates with other factors to regulate gene transcription (10,11). In Th17 cells, loss of BATF is known to cause diminished chromatin accessibility and transcription factor recruitment at lineage-specifying loci (11-13). *Batf* knockout mice were shown to have normally differentiated Th1 and Th2 cells but lacked differentiated Th17 cells. When stimulated toward Th17 lineage in vitro, *Batf* knockout T cells produced normal levels of IL-2, interferon γ, and IL-10 but Q8 reduced level of IL-17 (14). Therefore, the prolonged production of IL-17 by Th17 cells depends on the synergistic actions of RORγt and BATF-JUNB transcription factors (14). Whether *BATF*-dependent Th17 cells play a role in prostate cancer, however, remains unknown. To understand the role of Th17 cells in prostate cancer, a mouse model of autochthonous prostate cancer is essential.

In the present study, we crossed *Batf* knockout mice with *Pten*-deficient mice, an established prostate cancer mouse model, to determine the specific role of *Batf*-dependent Th17 cells in prostate cancer initiation and progression.

## Methods

### Statistics

Statistical analysis was performed using R (R Foundation for Statistical Computing, Vienna, Austria). The genitourinary (GU)-bloc weights were compared using the *t* test and analysis of variance. The *t* test was used to analyze the remaining data. All in vitro experiments were repeated at least 3 times. Unless otherwise indicated, data are presented as mean (95% confidence interval [CI]). See Supplementary Methods for additional methods; primer sequences are in Supplementary Table 1 (available online), and antibody information is in Supplementary Table 2 (available online).

## Results

### *Batf‒* mice developed smaller prostate tumors than *Batf+* mice

**Figure 1.**
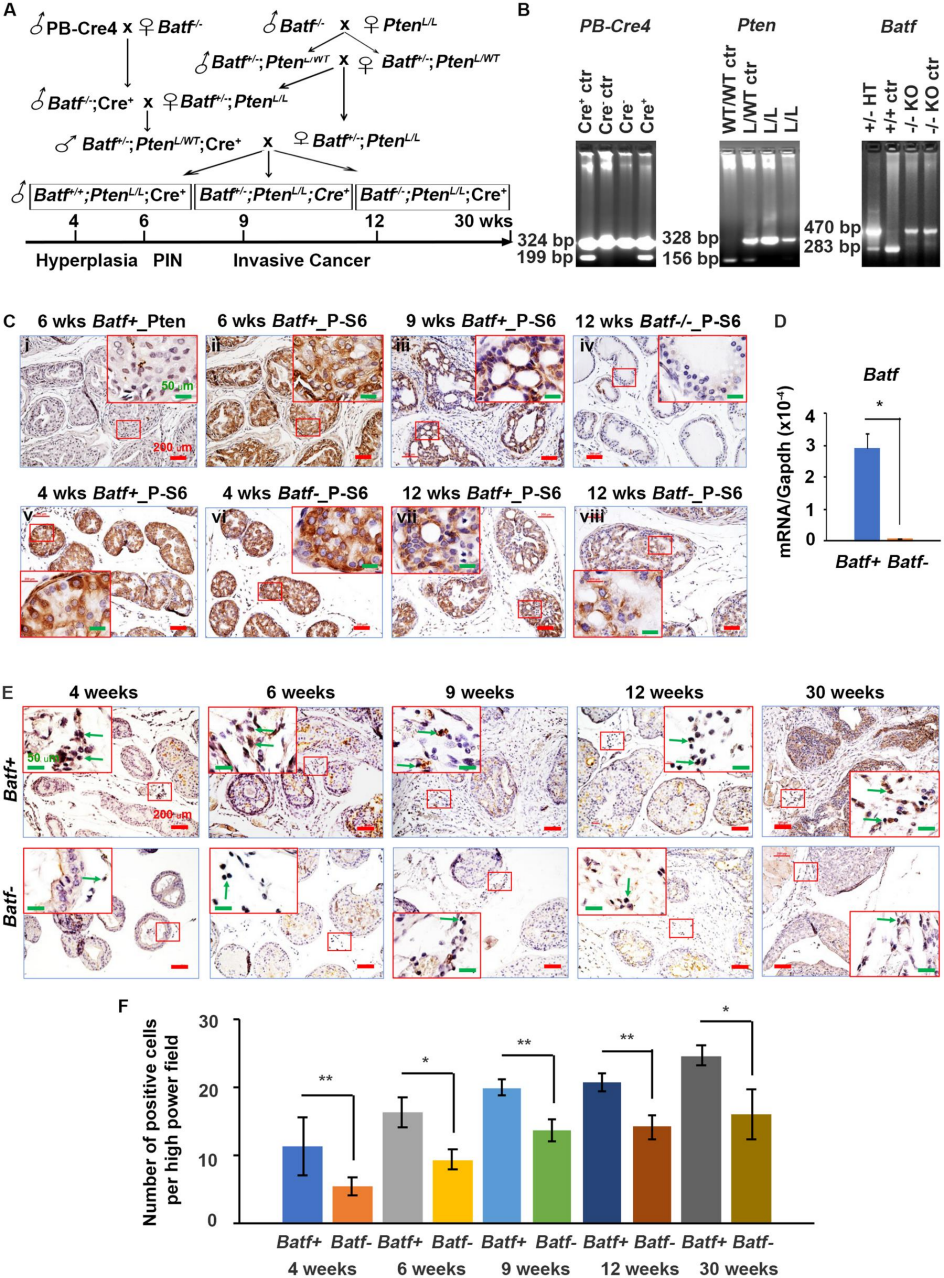
The confirmation of animal models. **A)** Strategy of animal breeding. **B)** Gel images showing representative polymerase chain reaction genotyping. **C)** Representative immunohistochemistry of Pten and P-S6 in the dorsal prostate lobes of mice at 4, 6, 9, and 12 weeks of age. Ci and Cii show immunohistochemical staining for Pten and P-S6 in consecutive sections of prostate tissue from 6-week-old *Batf+* mice, indicating *Pten* loss and activation of P-S6. Ciii shows immunohistochemistry for P-S6 in prostate tissue from a 9 week-old *Batf+* mouse, indicating activation of P-S6. Civ shows immunohistochemistry for P-S6 in the prostate tissue of a 12-week-old *Batf* knockout mouse with *Pten* wild type (named *Batf ^-/-^*), indicating that *Pten* wild-type prostate tissue has no activation of P-S6. Cv-Cviii show immunohistochemistry for P-S6 in 4- and 12-week-old *Pten*-deficient mice with or without *Batf* expression. Original magnification, ×100 (scale bar, 200 µm); inserts ×400 (scale bar, 50 µm). **D)** Quantitative polymerase chain reaction results for *Batf* in the prostate tissues of 30-week-old mice. **E)** Representative immunohistochemistry for Batf in the immune cells of *Batf+* and *Batf-* mice stroma. Original magnification, ×100 (scale bar, 200 µm); inserts, ×400 (scale bar, 50 µm). **F)** Number of Batf-positive immune cells per high-power field. Data are mean (95% confidence interval); n = 3 mice/group. *P <* *.05, **P <* *.01.

Figure 1, A shows the breeding strategy. The male pups were genotyped at 3 weeks of age (Figure 1, B). Immunohistochemical staining confirmed loss of *Pten* (Figure 1, Ci) and P-S6 (downstream target of the P-Akt/mTOR pathway) activation (Figure 1, Cii) in the prostatic epithelium of 6-week-old *Pten*-deficient mice with *Batf* expression (*Batf*+) in consecutive sections. The prostatic epithelium of other groups of mice either positive or negative for *Batf* all had activated P-S6 (Figure 1, Ciii and 1, Cv-Cviii), as previously reported (8,15-17). *Pten* wild-type mice had inactivated P-S6 (Figure 1, Civ). The prostates of *Batf* knockout mice deficient in *Pten* (*Batf-*) had undetectable *Batf* messenger RNA (mRNA) (Figure 1, D). *Batf*-positive cells in the prostate stroma were significantly reduced in *Batf-* mice compared with *Batf*+ mice at different time points (Figure 1, E and F), and the immune cell number increased with the tumor progression (Figure 1, F).

**Figure 2.**
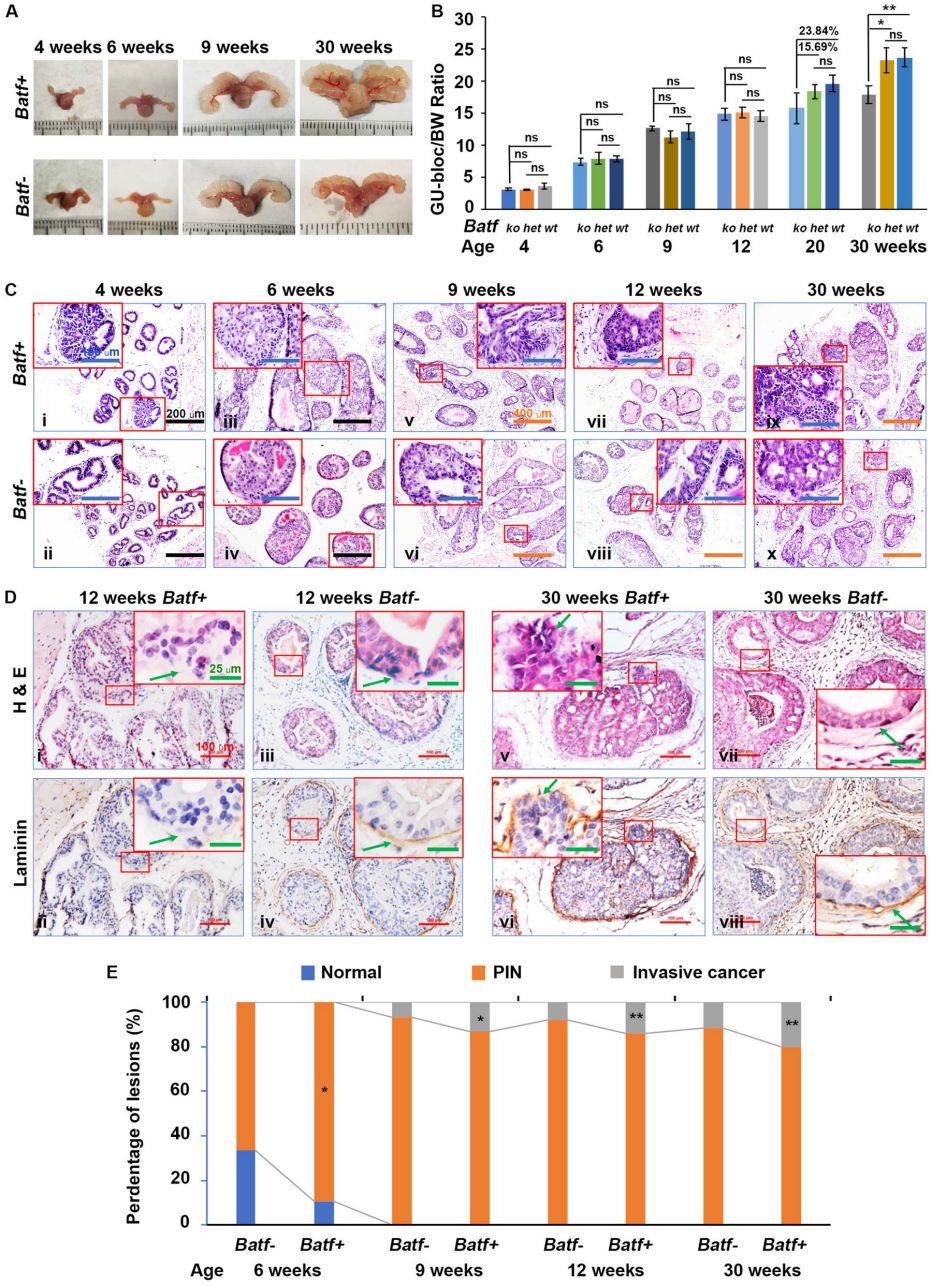
*Batf* knockout decreased prostate tumor growth and the formation of invasive prostate adenocarcinoma. **A)** Representative photograph of the genitourinary (GU)-blocs. **B)** The ratios of GU-bloc weights normalized by body weights (BWs) over the time course. **C)** Representative Hematoxylin and Eosin (H&E)-stained dorsal prostate lobes at 4, 6, 9, 12, and 30 weeks of age. Original magnification, Ci-Civ, ×200 (scale bar, 200 µm); Cv-Cx, ×100 (scale bar, 400 µm); inserts, ×400 (scale bar, 100 µm). **D)** Representatives of H&E and laminin staining in consecutive prostate sections of 12- and 30-week-old mice. Arrows in the inserts indicate the fibromuscular stroma or the front edges of invasion where the fibromuscular stroma is absent. Original magnification, ×100 (scale bar, 100 µm); inserts, ×400 (scale bar, 25 µm). **E)** Percentages of normal, prostatic intraepithelial neoplasia (PIN), and invasive adenocarcinomas. *P <* *.05, **P <* *.01.

The GU-bloc weight is proportional to the prostate weight and is often used to represent the tumor burden (18,19). Figure 2, A shows the GU-blocs of mice at 4, 9, and 30 weeks of age. At 30 weeks, the GU-blocs of *Batf*+ mice weighed significantly more than those of *Batf-* mice, but there was no noticeable difference at 4, 6, and 9 weeks of age. The 2 groups had no significant differences in body weight. The GU-bloc weight of each mouse was normalized by their corresponding body weight. Before 20 weeks, there was no significant difference between *Batf*+ and *Batf-* mice. At 20 weeks, however, the GU-bloc to body weight ratio of *Batf* wild-type and heterozygous (*HET*) mice (all *Pten-*null) was 23.84% and 15.69% heavier than that of *Batf-* mice (Figure 2, B). The body weight, GU-bloc, and GU-bloc to body weight ratio of *Batf* knockout mice was not different from *Batf^+/+^* (*Batf* wild-type with Pten wild-type) mice (Supplementary Figure 4, A, available online).

### *Batf* knockout decreases the formation of prostatic intraepithelial neoplasia and invasive adenocarcinoma

*Pten*-deficient mice develop epithelial hyperplasia at 4 weeks of age, prostatic intraepithelial neoplasia (PIN) at 6 weeks of age, and invasive adenocarcinoma at 9 weeks of age (20). We found that *Batf-* mice developed reduced epithelial hyperplasia at 4 weeks of age and PIN (66%) at 6 weeks of age compared with *Batf*+ mice (89%) (Figure 2, Ci-Cii and Ciii-Civ; Figure 2, E). At 9 weeks of age*,* 13% of prostate glands had invasive adenocarcinomas, and 87% of glands had PIN in *Batf+* mice. In contrast, only 7% of prostate glands had invasive adenocarcinoma, and 93% had PIN in *Batf-* mice (Figure 2, Cv-Cvi; Figure 2, E). At 12 weeks of age, 14% of prostate glands developed invasive adenocarcinoma, and 86% developed PIN in *Batf*+ mice compared with 8% of invasive adenocarcinoma and 92% PIN in *Batf-* mice (Figure 2, Cvii-Cviii; Figure 2, E). At 30 weeks of age, 20% of prostate glands presented as invasive adenocarcinoma in *Batf*+ mice, and only 12% of prostate glands showed invasive adenocarcinoma in *Batf-* mice, with the remaining 88% still presenting with PIN (Figure 2, Cix-Cx; Figure 2, E). The invasion of the basement membrane and the fibromuscular layer was confirmed by laminin staining (Figure 2, D). The percentages of PIN and invasive adenocarcinomas differed significantly between *Batf+* and *Batf-* mice at 6, 9, 12, and 30 weeks of age (Figure 2, E).

### *Batf* knockout reduced cellular proliferation, increased apoptosis, and decreased angiogenesis and inflammatory cell infiltration

**Figure 3.**
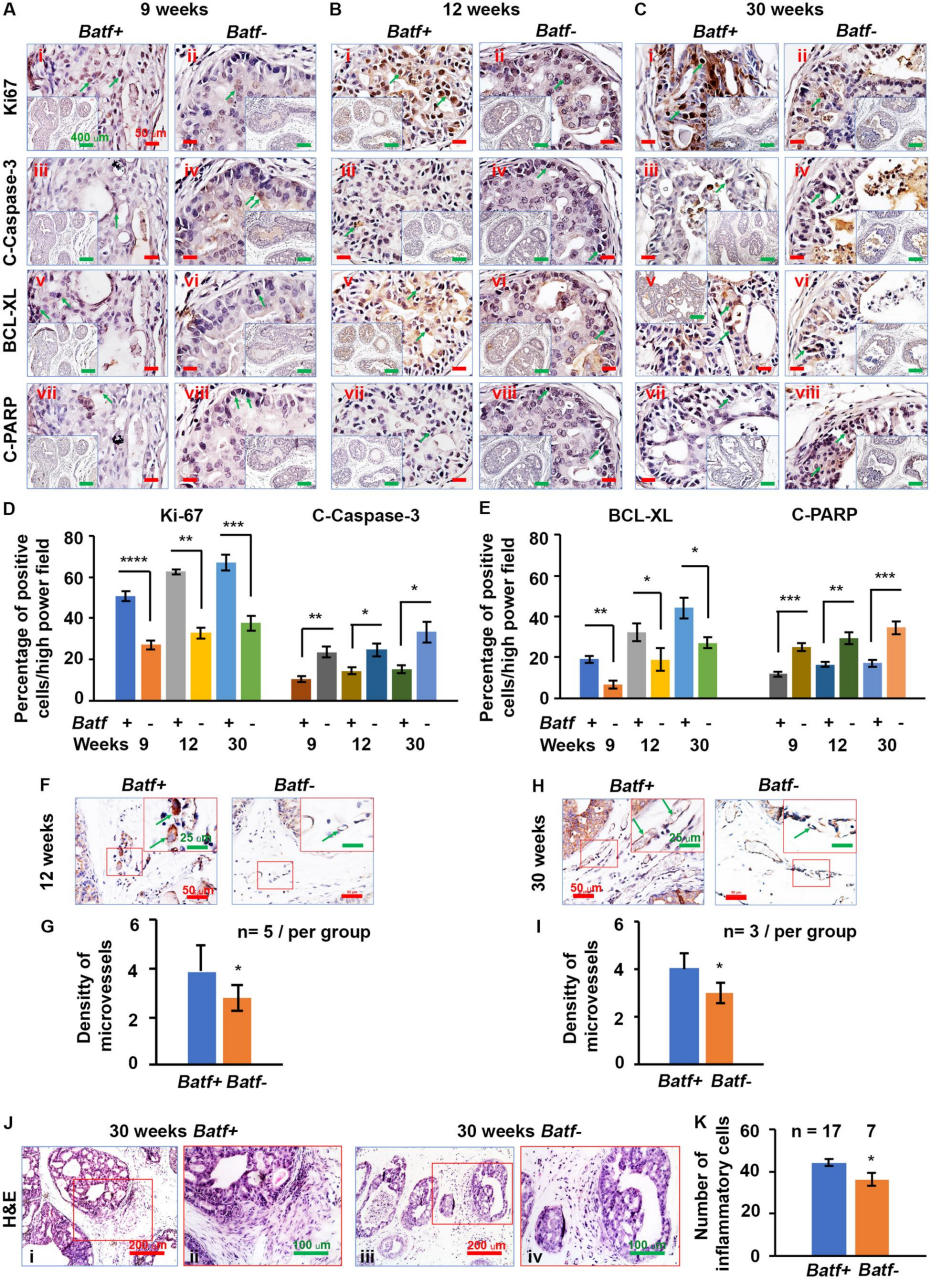
*Batf* knockout decreased cellular proliferation, increased apoptosis, and reduced angiogenesis in prostate tissues. **A-C)** Ki-67, C-Caspase-3, BCL-XL, and C-PARP staining in the prostate tissue of mice at 9, 12, and 30 weeks of age; arrows indicate the positive cells. Original magnification, ×400 (scale bar, 50 µm); inserts, ×100 (scale bar, 400 µm). **D, E)** Percentages of positive cells for Ki-67 and C-Caspase-3. (**D**) and BCL-XL and C-PARP (**E**) in dorsal lateral prostate lobes. *P <* *.05, **P <* *.01, ***P <* *.001, ****P <* *.0001. **F, H)** Representative CD31 staining for new blood vessels. Original magnification, ×200 (scale bar, 50 µm); inserts, ×400 (scale bar, 25 µm); arrows indicate new blood vessels. **G, I**) The density of microvessels. **J)** Representative Hematoxylin and Eosin (H&E)-stained sections in the prostate tissue of 30-week-old mice. Original magnification, Ji and Jiii, ×200 (scale bar, 200 µm); Jii and Jiv, ×400 (scale bar, 100 µm). **K)** Number of inflammatory cells counted on H&E-stained sections. *P <* *.05.

*Batf* knockout mice had altered prostate epithelial morphology compared with normal prostate, and *both Batf-* and *Batf^-/-^* mice showed reduced prostate-specific epithelial and stem cell markers. To understand why *Batf+* mice developed larger prostate tumors than *Batf-* mice, we assessed cellular proliferation and apoptosis by performing Ki-67 and apoptotic markers staining on consecutive sections of the prostate. We found more Ki-67–positive cells in the prostates of *Batf+* mice than in *Batf-* mice at 4, 6, 9, 12, and 30 weeks (Supplementary Figure 1, Ai-Avi, available online; Figure 3, Ai-Cii). In addition, there were more BCL-XL–positive antiapoptotic cells in the prostates of *Batf+* mice than in *Batf-* mice (Figure 3, Av-Cvi). These differences were significant (Figure 3, E). In contrast, apoptotic cells were reduced, as revealed by staining apoptotic markers Cleaved Caspase-3 (C-Caspase-3) (Figure 3, Aiii-Civ; Supplementary Figure 1, Bvii-Bxii, available online) and Cleaved PARP (C-PARP) (Figure 3, Avii-Cviii) in adjacent consecutive sections. These differences were significant (Figure 3, D and E). Furthermore, in the prostate stroma, we found that *Batf-* mice had significantly reduced microvessels compared with *Batf+* mice by immunohistochemical staining of CD31 at 12 and 30 weeks (Figure 3, F and H), and these differences were significant (Figure 3, G and I). More interestingly, we found that inflammatory cell infiltration was significantly reduced in *Batf-* compared with *Batf+* mice prostate stroma (Figure 3, J-K). *Batf-* or *Batf^-/-^* mice had reduced prostate tissue–specific epithelial markers compared with *Batf+* or *Batf^+/+^* mice, and *Batf* knockout mice had morphologic changes in prostate epithelia, even in *Pten* wild-type prostates (Supplementary Figures 2 and 3, available online).

### *Batf‒* mice had decreased expression of Th17-related cytokines and transcription factors; disrupted Th17 to Treg ratio in splenocytes, serum, and prostate; and reduced CD4^+^ and IL-17^+^ cells in prostate stroma

**Figure 4.**
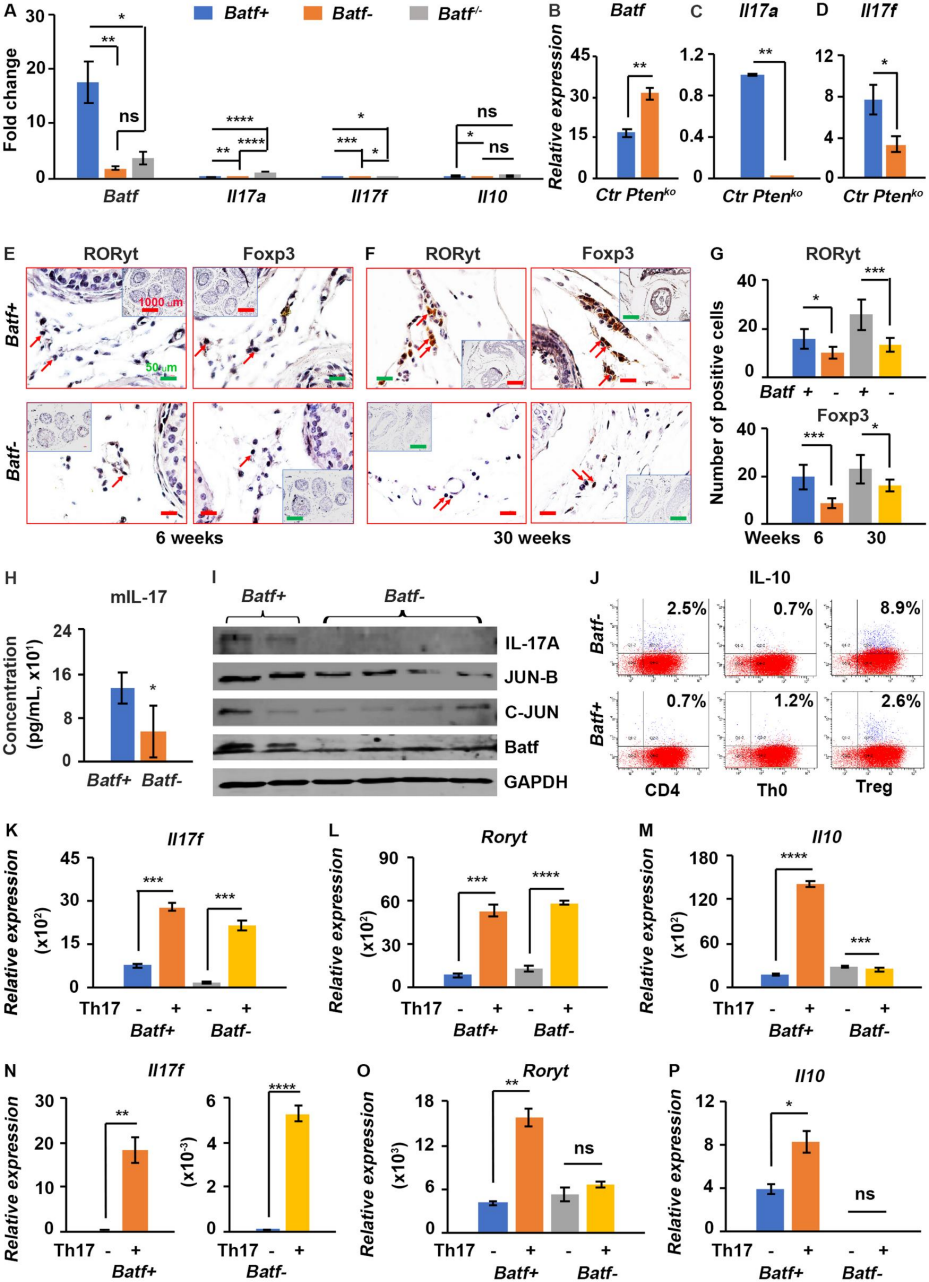
*Batf* knockout decreased the expression of Th17-related cytokines, transcription factors, and AP-1 family members in the prostate, serum, and splenocytes of *Pten*-deficient and *Pten* wild-type mice. **A)** Quantitative polymerase chain reaction results for *Batf*, *Il17a, Il17f*, and *Il10* in the prostate tissue of 12 week-old *Batf+*, *Batf-*, and *Batf^-/-^* (*Batf* knockout with *Pten* wild-type) mice. *Il17a* was significantly reduced in *Pten*-deficient mice, even lower than in *Batf^-/-^* normal mice. **B-D)** Quantitative polymerase chain reaction results for *Batf, Il17a,* and *Il17f* in the prostate tissue of 9 week-old *Pten*-deficient mice (*Pten^ko^*) vs age-matched *Pten* wild-type (Ctr) mice. *Batf* was significantly increased, but Il17a and Il17f were reduced. **E, F)** Representative immunohistochemical staining for RORγt and Foxp3 in the prostate stroma of mice at 6 and 30 weeks of age. Original magnification, ×400 (scale bar, 50 µm); inserts, ×100 (scale bar, 1000 µm). **G)** Number of positive cells for RORγt (upper panel) and Foxp3 (lower panel). *P <* *.05; ***P <* *.001. **H)** Enzyme-linked immunosorbent assay results for IL-17A protein in mouse serum. **I)** Immuno blot results in CD4^+^ T cells isolated from mouse spleen. **J)** Naive CD4^+^ T cells were ex vivo cultured under Treg differentiation conditions for 4 days, stimulated by Phorbol-12-myristate-13-acetate (PMA) and ionomycin for 6 hours, and then flow cytometry was performed. Only CD4^+^ T cells were gated, and interleukin 10 (IL-10)–expressing CD4^+^ T cells were analyzed. **K-M)** Quantitative polymerase chain reaction results for *Il17*, *RORγt,* and *Il10* in splenocytes cultured under Th17 differentiation condition plus IL-23 for 72 hours. Th17‒, Th0 condition, Th17+, Th17 condition. **N-P)** Quantitative polymerase chain reaction results for *Il17*, *RORγt,* and *Il10* in prostate tissue cultured under Th17 differentiation condition plus IL-23 for 72 hours. *P <* *.05, **P <* *.01, ***P <* *.001, ****P <* *.0001; ns = not significant.

In *Batf-* mouse prostate, *Batf*, *Il17a, Il17f*, *and Il10* mRNAs were significantly reduced compared with *Batf*+ (Figure 4, A; Supplementary Figure 4, B-E, available online). In *Batf* knockout mice, *Batf*, *Il17a*, and *IL17f* were also significantly reduced compared with *Batf^+/+^* mice, but *Il10* mRNA was significantly higher than *Batf^+^*^/+^ mice (Supplementary Figure 4, F-I, available online). Compared with *Pten* wild-type mice, *Pten*-deficient mice had significantly increased *Batf* but reduced *Il17a* and *Il17f* mRNAs (Figure 4, B-D). In *Batf-* mouse prostate stroma, RORγt–positive and Foxp3–positive cells were reduced compared with *Batf+* mice (Figure 4, E, F), and these differences were significant (Figure 4, G). In *Batf-* mouse serum, IL-17 protein was significantly reduced compared with *Batf*+ mice (Figure 4, H). In *Batf-* mouse CD4^+^ T cells, IL-17, Jun-B, and C-Jun were reduced compared with *Batf*+ mice (Figure 4, I). However, IL-10 expression increased in CD4^+^ T cells and Treg cells compared with *Batf*+ mice (Figure 4, J). Further, when splenocytes were cultured under Th17 differentiation conditions, *Il17f* and *RORγt* mRNAs were significantly increased in Th17 cells compared with naive T cells from both *Batf*+ and *Batf-* mice, but *Il10* mRNA was significantly reduced in *Batf-* mice (Figure 4, K-M). In addition, when prostate tissue was cultured under Th17 differentiation conditions, only *Il17f* mRNAs were significantly increased in both *Batf*+ and *Batf-* mice (Figure 4, N), and *RORγt* was increased only in *Batf*+ but not in *Batf-* mice (Figure 4, O). The *Il10* mRNAs were increased in *Batf*+ but not in *Batf-* mice (Figure 4, P). In prostate stroma, CD4^+^ and IL-17^+^ cells but not CD8^+^ cells were significantly reduced in *Batf-* mice compared with *Batf+* mice; however, IL-10^+^ cells were significantly increased (Supplementary Figure 5, available online). These results indicate that *Batf* affects Th17-related cells and the Th17 to Treg ratios in *Pten*-deficient conditions.

### *Batf* knockout decreased nuclear factor–κB activity, and CD4^+^ Th17 cell conditioned media from *Batf‒* mice reduced nuclear factor–κB activity in human prostate cancer cells

**Figure 5.**
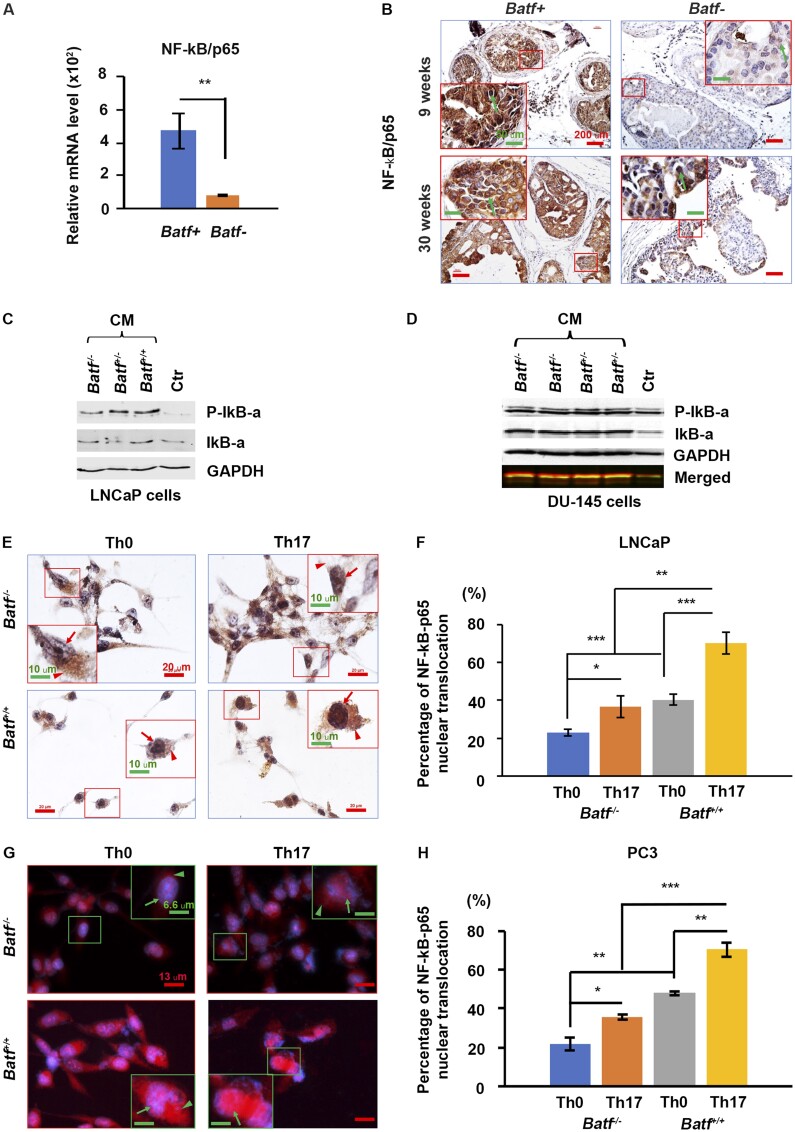
*Batf* knockout decreased nuclear factor–κB (NF-κB) activation. **A)** Quantitative polymerase chain reaction results for messenger RNA (mRNA) level of NF-κB–P65 in prostate tissues. Data are represented as mean (95% confidence interval), n = 3 animals per group, **P <* *.01. **B)** Representative prostate sections stained for NF-κB–P65 in mouse prostate tissues at 9 and 30 weeks of age. Original magnification, ×100 (scale bar, 200 µm); inserts, ×400 (scale bar, 50 µm); arrows indicate positive cell nuclear translocation. **C-D)** Representative Immuno blot results for NF-κB signaling in prostate cancer cell lines after exposure to conditioned media (CM) from *Batf^+/+^* or *Batf^-/-^* mouse naive CD4^+^ T cells (Th0) or differentiated Th17 cells for 48 hours. **E)** Representative immunohistochemistry for NF-κB-p65 in LNCaP (human prostate cancer cell line) cells after exposure to CM from *Batf^+/+^* or *Batf ^-/-^* mouse naive CD4^+^ T cells (Th0) or differentiated Th17 cells for 48 hours. Original magnification, ×400 (scale bar, 20 µm); insert, ×800 (scale bar, 10 µm). **F)** Percentage of NF-κB-P65 nuclear translocation per high-power field. *P <* *.05, **P <* *.01, ***P <* *.001. **G)** Representative immunofluorescence for NF-κB–P65 in PC3 (human prostate cancer cell line) cells after exposure to CM from *Batf^+/+^* or *Batf^-/-^* mouse naive CD4^+^ T cells (Th0) or differentiated Th17 cells for 48 hours. Original magnification, ×600 (scale bar, 13 µm); insert, ×1200 (scale bar, 6.6 µm). **H)** Percentage of NF-κB–P65 nuclear translocation per high-power field. *P <* *.05, **P <* *.01, ***P <* *.001.

We examined nuclear factor–κB (NF-κB) signaling in mouse prostate tissues to understand why *Batf-* mice had reduced cellular proliferation and increased apoptosis. We found that *Batf-* mouse prostates had significantly reduced NF-κB**/**p65 mRNAs compared with *Batf*+ mice (Figure 5, A). In addition, *Batf-* mice had reduced NF-κB**/**p65 protein nuclear translocation compared with *Batf*+ mice (Figure 5, B). Further, we analyzed NF-κB signaling activation in prostate cancer cells after exposure to the CD4^+^ T-cell–secreted factors (conditional media) (21) from *Batf^+/+^, Batf^+/-^* (mice have the *Batf* gene heterozygous with Pten wild-type), and *Batf* knockout mice for 48 hours. We found that the conditional media from *Batf^+/+^* and *Batf^+/-^* mice induced prostate cancer cells’ NF-κB activation, with increased cytosol P-IκB-α protein levels compared with conditional media from *Batf *knockout mice (Figure 5, C, D). Furthermore, the conditional media from both Th17 cells and Th0 cells of *Batf^+/+^* mice induced prostate cancer cells’ NF-κB nuclear translocation compared with that from *Batf* knockout mice, and more NF-κB nuclear translocation was found from those exposed to factors from differentiated Th17 cells than from Th0 cells (Figure 5, E, G). These differences were significant (Figure 5, F, H).

### *Batf* knockout reduced IL-23-IL-23R signaling and macrophages; Batf can bind to AP-1 motifs in the *IL-23R* gene promoter region

**Figure 6.**
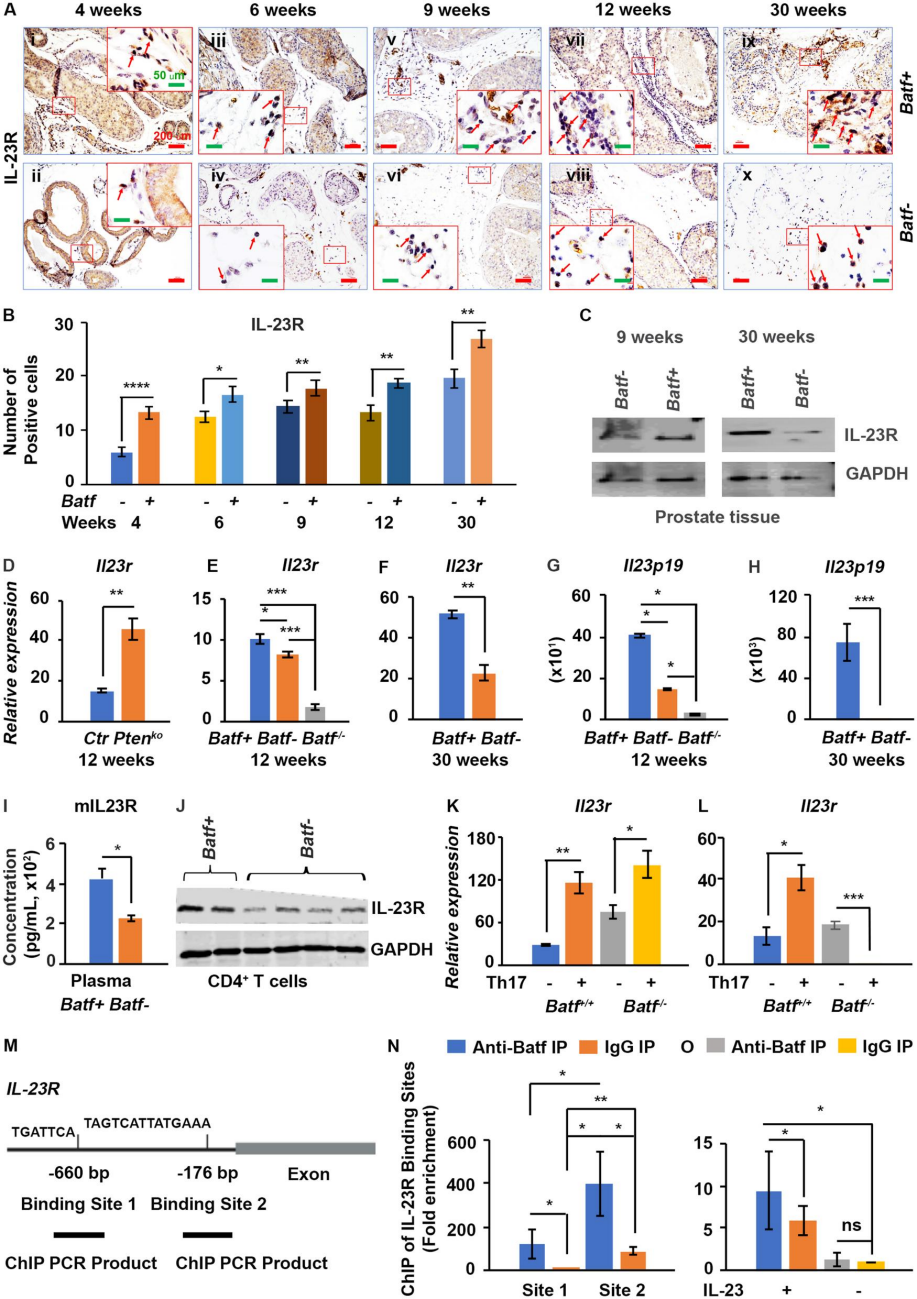
*Batf* knockout decreased interleukin 23 (IL-23)-IL-23R signaling in *Pten*-deficient mice, and Batf binds to the motifs of the mouse *IL-23R* gene promoter region. **A)** Representative immunohistochemistry for IL-23R–positive immune cells of *Batf+* and *Batf-* mice stroma at different ages. Original magnification, ×100 (scale bar, 200 µm); inert, ×400 (scale bar, 50 µm). **B)** Numbers of IL-23R–positive cells per high-power field. Data are mean (95% confidence interval), n = 3 mice/group. *P <* *.05, **P <* *.01. **C)** Representative Western blot result for IL-23R in prostate tissue of *Batf+* and *Batf-* mice at 9 and 30 weeks of age. **D)** Quantitative reverse transcriptase–polymerase chain reaction (PCR) results of *Il23r* in the prostate tissue of 12-week-old *Pten* wild-type mice (Ctr) and *Pten*-null mice. **E)** Quantitative reverse transcriptase–PCR results of *Il23r* in the prostate tissue of 12-week-old *Batf+* and *Batf- (Pen*-null*)*, and *Batf* knockout (*Pten* wild-type) mice. **F)** Quantitative reverse transcriptase–PCR results of *Il23r* in the prostate tissue of 30-week-old *Batf+* and *Batf-* mice. **G)** Quantitative reverse transcriptase–PCR results of *Il23p19* in the prostate tissue of 12-week-old *Batf+* and *Batf- (Pten*-null*)*, and *Batf* knockout (*Pten* wild-type) mice. **H)** Quantitative reverse transcriptase–PCR results of *Il23p19* in the prostate tissue of 30-week-old *Batf+* and *Batf-* mice. **I)** Mouse IL-23R levels in the plasma of *Batf+* and *Batf-* mice. **J)** Representative Western blot for IL-23R in CD4^+^ T cell lysates from *Batf+* and *Batf-* mice. **K-L)** Quantitative reverse transcriptase–PCR results for *Il23r* messenger RNA in *Batf+* and *Batf-* mouse splenocytes (**K**) or in ex vivo cultured mouse prostate tissue (**L**) in Th17 differentiation conditioned media for 72 hours. *P <* *.05, **P <* *.01, ***P <* *.001. **M)** IL-23R binding sites and chromatin immunoprecipitation (ChIP) quantitative PCR product information. **N, O)** The results of ChIP assays were measured by quantitative PCR. Enrichment of *IL23R* binding sites using anti-Batf rabbit polyclonal antibody on sheared chromatin from mouse splenocytes. Normal rabbit immunoglobulin G (IgG) was used as a negative immunoprecipitation (IP) control. The purified DNA was analyzed on the Bio-Rad CFX Opus 96 Real-Time PCR System, with optimized primers for the promoter region of the AP-1 motifs in the *IL23R* gene. Data are presented as fold enrichment of the antibody signal vs the negative control IgG without the IL-23 treatment group, calculated using the comparative Ct method (also referred to as the 2^-△△^^Ct ^method). Data are mean (95% confidence interval) (n = 3) of 3 independent experiments. *P <* *.05, **P <* *.01, ns = not significant difference compared with the corresponding IgG IP groups.

*Pten*-deficient mice had reduced *Il17* but increased *Batf*; we wondered whether the reduced tumor initiation and progression resulted from other factors. We compared IL-23-IL-23R signaling between *Batf+* and *Batf-* mice and found that the IL-23R-postive (IL-23R^+^) cells in the prostate stroma were significantly reduced in *Batf-* compared with *Batf+* mice (Figure 6, A), and their differences were significant (Figure 6, B). Furthermore, we found that IL-23R protein level was reduced in *Batf-* prostate lysates compared with *Batf+* mice (Figure 6, C). Interestingly, *Pten*-deficient mice had significantly increased mRNAs of *Il23r* compared with *Pten* wild-type control mice; this finding is consistent with the Batf expression (Figure 6, D). Furthermore, *Il23r* and *Il23p19* mRNAs were significantly reduced in *Batf- or Batf^-/-^* mice compared with *Batf+* or *Batf^+/+^* mice (Figure 6, E-H; Supplementary Figure 4, J and K, available online). Moreover, *Batf-* mice had significantly reduced IL-23R protein levels in the plasma and CD4^+^ T cells (Figure 6, I and J). Under Th17 conditions, splenocytes from both *Batf*+ and *Batf-* mice produced significantly increased *Il23r* compared with naive cells (Figure 6, K), but the *Batf-* mouse prostate tissue had significantly decreased *Il23r* production under the Th17 condition compared with *Batf*+ mice (Figure 6, L).

IL-23 acts through IL-23R to maintain Th17 cells (22). The *IL-23R* gene is expressed in IL-23–stimulated CD4^+^ T cells. Previous studies indicated that in CD4^+^ T cells, IRF4 can cooperate with AP-1 complexes to bind to AP1-IRF4 composite (5ʹ-TGAnTCA/GAAA-3ʹ) motifs. BATF-JUN family protein complexes cooperate with IRF4 to secure these motifs in preactivated CD4^+^ T cells stimulated with IL-21– and Th17-differentiated cells (11). To investigate the *IL-23R* gene potential cooperative binding by the *BATF* complex, we identified strong IL-23R binding sites containing motifs adjacent to AP-1 motifs. We selected these sites and confirmed the co-localization of *BATF* by chromatin immunoprecipitation assay to the binding sites in splenocytes stimulated with IL-23 (Figure 6, M-O). We further transfected *Batf* to mouse prostate cancer cells and confirmed the binding of *Batf* to the *IL-23R* promoter region (Figure 8, F). In addition, we found that IL-23 stimulated *Il23r* and *Batf* expression in splenocytes (Figure 8, E).

**Figure 7.**
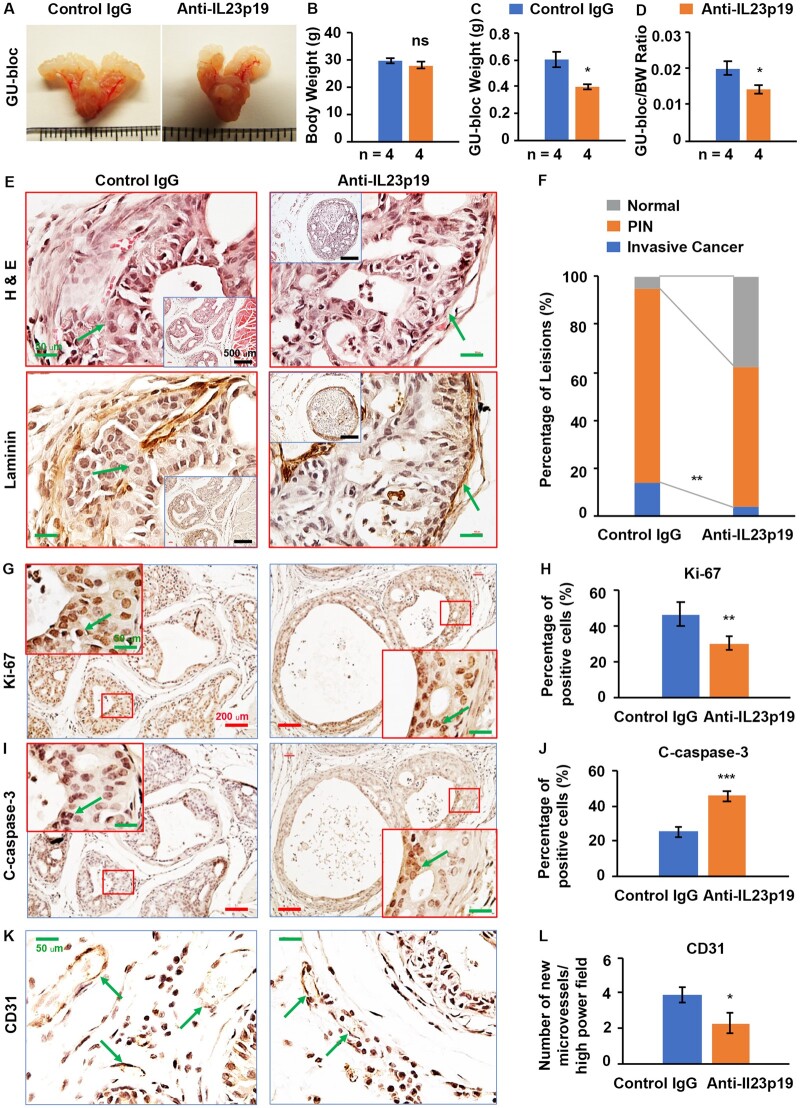
Anti–interleukin 23 (IL-23)p19 antibody treatment decreased prostate adenocarcinoma in *Pten-*deficient mice. **A)** Representative photographs of genitourinary (GU)-blocs. **B)** Body weight (BW). **C)** GU-bloc weight. **D)** GU-bloc to BW ratio; the number of animals in each group is shown under the abscissa. *P <* *.05 compared with control mice. **E)** Representative sections of dorsal prostatic lobes were stained with Hematoxylin and Eosin (H&E) and subjected to immunohistochemistry for laminin in consecutive sections for both groups. Arrows indicate an invasive site in control mice and a noninvasive site in IL-23p19 antibody–treated mice. Original magnification, ×400 (scale bar, 50 µm); inserts, ×100 (scale bar, 500 µm). **F)** Percentages of prostatic intraepithelial neoplasia (PIN) and cancer in dorsal prostatic lobes of anti–IL-23p19 and anti–immunoglobulin G (IgG)–treated mice. The number of animals in each group is shown under the abscissa. *P <* *.05 compared with control mice. **G, I)** Representative prostate sections stained for Ki-67 and apoptosis (C-caspase-3) in consecutive sections in anti–IL-23p19 antibody and anti-IgG–treated mice. Arrows indicate the positive cells. Original magnification, ×100 (scale bar, 200 µm); inert, ×400 (scale bar, 50 µm). **H)** Percentages of Ki-67–positive cells in mouse prostates. Data are presented as mean (95% confidence interval) (n = 3 animals per group), **P <* *.01. **J)** Percentages of apoptotic cells in anti–IL-23p19 antibody and anti-IgG–treated mouse prostates. Data are represented as mean (95% confidence interval) (n = 3 animals per group), ***P <* *.001. **K)** Representative prostate sections stained for CD31 in anti–IL-23p19 antibody and anti-IgG–treated mice. Arrows indicate the positive cells. **L)** Density of micro–blood vessels per high-power field in anti–IL-23p19 antibody and anti-IgG–treated mouse prostates. Data are represented as mean (95% confidence interval) (n = 3 animals per group), *P <* *.05.

Activated macrophages and dendritic cells mainly secrete IL-23. We therefore performed immunohistochemical staining in consecutive sections for macrophages, including its 2 subtypes, M1 and M2. Our results showed significantly reduced macrophages in *BATF‒* compared with *BATF+* mice (Supplementary Figure 6, A-F, available online) at different ages, and the cell numbers increased with tumor progression.

### Anti–IL-23p19 treatment prevents prostate adenocarcinoma progression in *Pten*-deficient mice and reduces IL-23–/Th17-related immune cells in mouse prostate stroma; recombinant mouse IL-23 treatment increases *Il23r* and *Batf* mRNA levels in mouse splenocytes

Anti–IL-23p19 antibodies are currently being evaluated in clinical trials for treating autoimmune diseases, and they are clinically well tolerated (23). To evaluate the therapeutic relevance of our findings, we assessed whether IL-23 inhibition by antibody blockade could prevent prostate adenosarcoma progression in *PTEN*-deficient mice. We found that prostate tumors in the IL-23p19 antibody treatment group were smaller than those in the immunoglobulin G (IgG) control treatment group (Figure 7, A). We did not find a difference between the body weights of the control group and treatment group (Figure 7, B). Further, the GU-bloc and the GU-bloc to body weight ratio were significantly reduced in the treatment compared with the control group (Figure 7, C and D). We also found that the microinvasive prostate adenocarcinoma was significantly reduced in the treatment group compared with the control group (Figure 7, E). In the treatment group, only 4.05% of prostate glands presented with microinvasive prostate adenocarcinoma. In contrast, 14.29% of prostatic glands showed microinvasive prostate adenocarcinoma in the control mice. The differences in the percentages of lesions were statistically significant between the 2 groups of mice (Figure 7, F).

**Figure 8.**
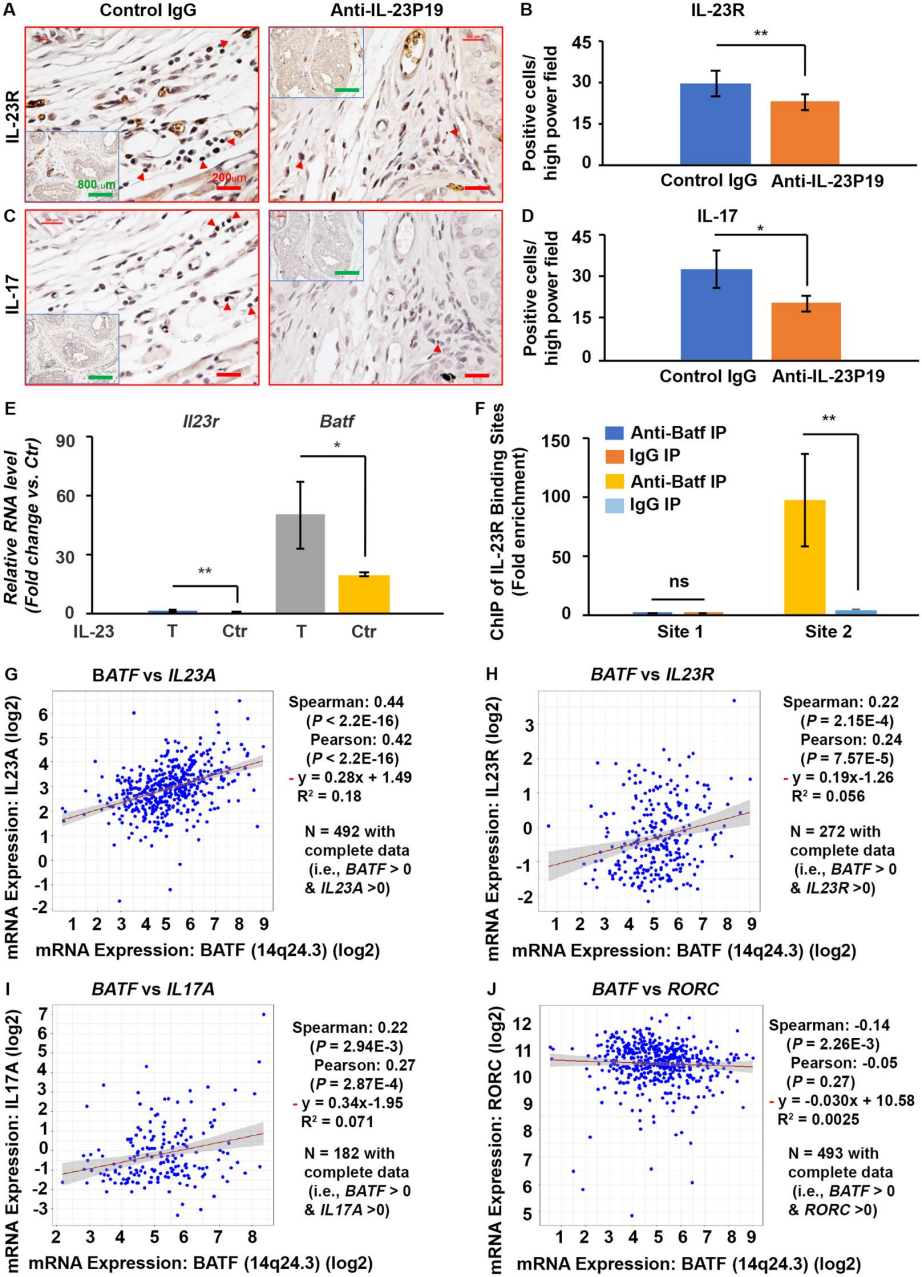
Anti–interleukin 23 (IL-23)p19 antibody treatment decreased IL-23R– and IL-17–positive cells in *Pten-*deficient mouse prostate stroma, recombinant mouse IL-23–treated *Pten* wild-type mouse splenocytes increased *Il23r* and *Batf* messenger RNA (mRNA) levels, Batf binds to IL-23R in mouse castration-resistant prostate cancer cell lines (MyC-CaP/CR), and *IL-23A* and *IL-23R* mRNA levels positively correlate with *BATF* mRNA levels in human prostate tumors. **A, C)** Representative immunohistochemical staining for IL-23R– and IL-17–expressing cells in consecutive sections of the prostate stroma of anti–IL-23p19 or control (Ctr) immunoglobulin G (IgG)–treated *Pten-*null mice. **B, D)** Number of positive cells per high-power field. *P <* *.05, **P <* *.01. **E)** Quantitative polymerase chain reaction results for *Il23r* and *Batf* mRNAs in mouse splenocytes treated with recombinant mouse IL-23 or control medium (without IL-23). **F)** The result of chromatin immunoprecipitation (ChIP) assays was measured by quantitative polymerase chain reaction. Enrichment of *IL23R* biding sites using anti-BATF rabbit polyclonal antibody on sheared chromatin from MyC-CaP/CR cells transfected with 4 μg pcDNA3.1-mBATF (Plasmid No. 34575, Addgene) for 48 hours. Normal rabbit IgG was used as a negative IP control. The purified DNA was analyzed on the Bio-Rad CFX Opus 96 Real-Time PCR System, with optimized primers for the promoter region of the AP-1 motifs in the *IL23R* gene. Data are presented as fold enrichment of the antibody signal vs the negative control IgG group, calculated using the comparative Ct method. Data are mean (95% confidence interval) (n = 3) of 3 independent experiments. **P <* *.01, ns = not significant difference compared with the corresponding IgG IP groups. **G-J)** The *BATF*, *IL23A*, *IL23R*, *IL17A*, and *RORC* mRNA expression, RSEM (batch normalized from Illumina HiSeq_RNASeqV2) were downloaded from the cBioPortal for genomics—Prostate Adenocarcinoma (The Cancer Genome Atlas Program [TCGA], Pan-Cancer Atlas), including 494 samples. **G)** Correlation between the mRNA levels of *IL23A* and *BATF*. **H)** Correlation between the mRNA levels of *IL23R* and *BATF*. **I)** Correlation between the mRNA levels of *IL17A* and *BATF*. **J)** Correlation between the mRNA levels of *RORC* and *BATF*. Sample size and the statistical analysis results are shown on each panel.

To reveal the underlying mechanisms, we assessed cellular proliferation and apoptosis. We found significantly reduced Ki-67–positive cells and increased apoptotic cells in antibody-treated prostates than in IgG-treated prostates (Figure 7, G-J; Supplementary Figure 7, A-H, available online). As IL-23 is an angiogenic factor (24), we assessed angiogenesis in mouse prostate tumors using immunohistochemical staining of CD31. We found significantly reduced new blood vessels in the antibody-treated prostate stroma compared with the control IgG-treated group (Figure 7, K and L). Furthermore, we found significantly reduced *Batf*-dependent IL-23R^+^ and IL-17^+^ cells (Figure 8, A-D) and IL-23-/Th17-related CD4^+^ cells, RORγt^+^, and IL-10^+^ cells (Supplementary Figure 8, A-J, available online). Still, the CD8^+^ and Foxp3-positive (Foxp3^+^) cells showed no significant decrease in anti–IL-23p19–treated prostate stroma compared with the control group. These results indicated that reduced angiogenesis and inflammatory immune cells in anti–IL-23p19–treated prostates contributed to the decreased prostate tumor burden in anti–IL-23p19–treated mice.

### *BATF* mRNA levels are positively correlated with IL-23A and IL23R in human prostate tumor tissue

To confirm that our findings have clinical significance, we analyzed the correlation of *BATF*, *IL23A*, *IL23R*, *IL17A*, and *RORC* mRNA expression in prostate adenocarcinoma (TCGA, Pan Cancer Atlas) through the cBioPortal for cancer genomics (25,26). We found that *BATF* mRNA expression has a closer correlation with *IL23A* (Spearman rank correlation = 0.44, *P* < 2.2E-16) (Figure 8, G) compared with the correlation between *BATF* and *IL23R* (Spearman rank correlation = 0.22, *P* = 2.15E-4) (Figure 8, H) and *BATF* and *IL17A* (Spearman rank correlation = 0.22, *P* = 2.94E-3) (Figure 8, I). *BATF* negatively correlated with *RORC* (Spearman rank correlation = ‒0.14, *P *= 2.26E-3) (Figure 8, J) in prostate adenocarcinoma.

## Discussion

Our study identified 2 significant phenotypic differences between *Batf+* and *Batf-* mice. *Batf-* mice developed smaller prostate tumors than *Batf+* mice at 30 weeks of age.

Moreover, *Batf-* mice developed significantly fewer invasive adenocarcinomas than *Batf+* mice. Our explanation for these phenomena is that *Batf-* mice had reduced expression of IL-17 and RORγt but increased expression of IL-10 and Foxp3, thus restoring the Th17/Treg balance. We have demonstrated that disruption of the Th17/Treg axis activates NF-κB signaling, increases cellular proliferation, and reduces apoptosis and angiogenesis (21). This study confirmed the inactivation of NF-κB signaling in the *Batf-* and *BATF^-/- ^*(*Batf* knockout mice with Pten wild-type) mice. In addition, *Batf* knockout changed prostate epithelial morphology, and tissue-specific epithelial and stem cell markers changed in *Batf-* and *Batf^-/-^* mice.

IL-23 promotes the production of inflammatory mediators such as IL-17 and IL-22 in target populations (27). This study discovered that *Batf-* mice showed significantly reduced IL-23-IL23R signaling. In *Batf-* mice, Il23p19 and IL-23R mRNA levels and protein decreased in the plasma, CD4^+^ T cells, and prostate tissue. In addition, the number of IL-23R^+^ and IL-17^+^ cells in the prostate stroma decreased significantly. We also found that BATF can directly bind to the promoter region of the *Il23R* gene in spleen and prostate cancer cells, indicating that *Il23R* is a target of BATF. Furthermore, *Batf* mice showed a significant reduction in macrophages, the primary cells responsible for secreting IL-23. Treatment with anti–IL23p19 antibody reduced tumor size and invasive adenocarcinoma compared with control IgG-treated mice. The mechanisms underlying these effects included reduced cellular proliferation, increased apoptosis, reduced angiogenesis, and decreased inflammatory cell infiltration, particularly of IL-23R^+^, IL-17^+^, CD4^+^, and RORγt^+^ cells, which are BATF dependent. Human prostate cancer TCGA data analysis also confirmed a correlation between BATF, IL-23A, and IL-23R but less so between BATF and RORC.

This study underscores the role of *Batf-*dependent Th17 cells in promoting prostate cancer initiation and progression in *Pten*-deficient mice. In these mice, *Pten* deletion triggers pathological changes; however, *Batf*-dependent Th17 cells disrupt the Th17/Treg axis, activating NF-κB signaling and the IL-23-IL-23R axis. Activating these pathways may increase cellular proliferation, inhibit apoptosis, enhance inflammation, and accelerate tumor progression.

In conclusion, our study indicates that *Batf*-dependent Th17 cells are crucial for developing prostate adenocarcinoma. Mechanistically, *Batf*-dependent Th17 cells promote the activation of NF-κB signaling and increase tumor-infiltrating IL-23R^+^ T cells and IL-23 production critical for establishing an IL-23–enriched tumor microenvironment. These results demonstrate that the BATF-IL-23R axis is a potential target for developing novel strategies for preventing and treating prostate cancer.

## Supplementary Material

djae120_Supplementary_Data

## Data Availability

The data underlying this article are available in the article and in its online supplementary material.
