# Supplementary material for: BATF-dependent Th17 cells act through the IL-23R pathway to promote prostate adenocarcinoma initiation and progression
Source: J Natl Cancer Inst. 2024 Jun 4;116(10):1598–611. doi: 10.1093/jnci/djae120 (PMC11461145; doi:10.1093/jnci/djae120)
Supplement: djae120_Supplementary_Data [file djae120_supplementary_data.pdf]

Supplementary Material

Supplementary Methods

Mice

The Animal Care and Use Committee of Tulane University approved the animal protocol. The Assurance Number: D16-00754 (A4499-01), effective 4/28/2022 through 3/31/2026. The Institutional Animal Care and Use Committee (IACUC) protocol ID is 1127. *Pten*$^{loxp/loxp}$*(Pten*$^{L/L}$*)* mice [1] (strain name: C; 129S4-*Pten*^{tm1Hwu}/J; genetic background: 129S4/SvJae*BALB/c) were obtained from the Jackson Laboratory (Bar Harbor, ME). PB-Cre4 mice [2] (strain name: B6.Cg-Tg(Pbsn-cre)4Prb; genetic background: B6.Cg) were obtained from Mouse Models of Human Cancers Consortium (MMHCC) of the National Cancer Institute. *Batf*$^{-/-}$ mice (strain name B6.129S-Batf$^{tm1.1Kmm}$/J; JAX stock #013758; genetic background: B6.Cg) were obtained from Jackson Laboratory and generated by Dr. Kenneth Murphy-Washington University School of Medicine using standard homologous recombination [3]. After crossbreeding *Pten*-deficient mice with *Batf*$^{-/-}$ mice, we got (*Batf*$^{+/+}$*;Pten*$^{L/L}$*;Cre*$^{+}$*), (Batf*$^{+/-}$*;Pten*$^{L/L}$*;Cre*$^{+}$*),* and *(Batf*$^{-/-}$*;Pten*$^{L/L}$*;Cre*$^{+}$*)* mice.

As *Batf*$^{+/+}$ and *Batf*$^{+/-}$ secrete similar levels of IL-17 [3], and our further results showed no difference in the onset and progression of prostate cancer (see Results section), we combined these two groups of mice into one group, named *Batf*$^{+}$, and for consistency, *Batf*$^{-/-}$ mice were named *Batf*$^{-}$. DNA was extracted from the tail biopsy for PCR genotyping as described [1, 2] [3] (see primer sequences in Supplementary Table 1).

Histopathology and inflammation examination

Mice were euthanized and weighed at 4, 6, 9, 12, and 30 weeks of age. The genitourinary (GU) blocs comprising the prostate lobes, seminal vesicles, ampullary glands, bladder, proximal ductus deferens, and proximal urethra were excised *en bloc* [5]. The GU-blocs were photographed, weighed with an empty bladder, and fixed as described [5]. Twenty-eight consecutive 4-μm sections of each prostate were cut, and four sections (from every seventh section) were H&E stained for histopathologic assessment in a blinded fashion according to the Bar Harbor Classification [5]. The prostatic glands were assessed under low- and high-power magnifications, and approximately 30 to 90 prostatic glands in each prostate were counted, with a total of over 500 prostatic glands in 10 mouse prostates per group. The number of inflammatory cells in the stroma space between the prostatic glands was counted in 6-10 high-power fields (x400 magnification) of each prostate lobe, including dorsal, lateral, ventral, and anterior prostatic lobes, and the average number of inflammatory cells per high-power field in more than three mouse prostates per genotype was compared.

Immunohistochemical and immunofluorescence staining

Immunohistochemical (IHC) staining and double immunofluorescent (IF) staining were performed as described previously [4, 6, 7]. The VECTSTAIN® ABC-HRP kits and DAB Substrate Kits (VECTSTAIN® Elite® ABC-HRP kit, Peroxidase (Goat IgG) (PK-6105), VECTSTAIN® Elite® ABC-HRP kit, Peroxidase (Rabbit IgG) (PK-6101), DAB Substrate Kit, Horseradish Peroxidase (HRP), with Nickel, (3,3'-diaminobenzidine) (SK-4100), Vector Laboratories, Inc., Newark, CA) were used according to the manufacturer's instructions. The antibodies used were rabbit anti-p-Akt (1:100) and mouse anti-PTEN (26H9, 1:50) (Cell Signaling Technology, Beverly, MA); anti-Ki-67 (1:1000, Millipore, Temecula, CA); anti-cleaved caspase-3 (1:100, C-caspase-3,

Cat #: 9664s), anti-cleaved caspase-7 (1:500, C-caspase-7, Cat #: 8438S), anti-cleaved caspase-9 (1:200, C-caspase-9, Cat #: 52873T), anti-cleaved-PARP (1:200, C-PARP, Cat No: 5625), and anti-BCL-XL (Cat No: 2764) from Cell Signaling Technology; rabbit anti-IL-17 (1:200, E19, sc-6077), rabbit anti-NOS2 (1:200, iNOS, N-20, sc-651), goat anti-arginase I (1:100, V-20, sc-18345), rabbit anti Sox9 (1:100, sc-20095 (H-90)), from Santa Cruz Biotechnology, Inc., Santa Cruz, CA; rabbit anti-CD31 (Ab28364, 1:50, Abcam, PLC., Cambridge, MA); rabbit anti-BATF (1:500; SAB4500122), rabbit anti-laminin (1:800; L9393), and anti-IL-23R (1:200; 06-1331) from Millipore Sigma. rabbit anti-CK8 (1:1000, Cat #: A01421), rabbit anti-CK19 (1:1000, Cat #: PB9715) from Boster Biological Technology; rabbit anti-F4/80 antibody (1:100, Cat #: ab100790) from Abcam. To quantify Ki-67-positive and cleaved caspase-3, 7, 9, c-PARP-positive cells, three animals from each genotype at 4, 6, 9, 12, and 30 weeks were randomly selected; three representative prostate sections from each animal were stained; approximately 300 cells per field of 6-10 high-power areas of each prostate lobe were counted; and the percentages of positive cells were calculated as the number of positive cells divided by the total number of cells. To quantify the immune cells in the prostate stroma, three animals from each genotype at 4, 6, 9, 12, or 30 weeks were randomly selected; three representative prostate sections from each animal were stained, and 6-10 high-power areas of sections were counted, and the number of positive cells was calculated. See detailed antibody information in Supplementary Table 2.

Preparation of spleen cells, $CD4^+$ T cells, Th17, Treg cell polarization, and $CD4^+$ T cell-secreted factors (conditioned media, CM)

Spleen cells were prepared from the spleens of mice [4]. Briefly, spleens were mashed and treated with ammonium-chloride-potassium (ACK) lysing buffer to remove red blood cells. The

cells were then passed through a 70 µm cell strainer to obtain individual cells, which were cultured in a complete culture medium for lymphocytes, RPMI 1640 medium (GenClone 25-506 RPMI 1640, with L-Glutamine and Phenol Red, 2000 mg/L D-Glucose, Cat#: 25-506, Genesee Scientific, A Life Science Company, EI Cajon, CA) containing 10% fetal bovine serum (PEAK SERUM, Cat #: PS-FB3, Bradenton, FL), 2 mM L-glutamine, 0.1 mM non-essential amino acids, 1 mM sodium pyruvate, 100 IU/mL penicillin/streptomycin, and 50 µM 2-mercaptoethanol (Cat #: 125472500, ThermoFisher Scientific). $CD4^+$ T cells from single-cell suspensions of murine spleens were enriched using a magnetic microbeads negative selection kit (Miltenyi Biotec Inc. CA, USA). Then, naïve $CD4^+$ T cells ($CD62L^+CD44^{io}$) were isolated using $CD4^+$ $CD62L^+$ T Cell Isolation Kit II mouse (Cat# 130-093-227, Miltenyi Biotec Inc.). Cells were activated with plate-bound anti-CD3 antibody (2 µg/mL, Biolegend) and CD28 antibody (2 µg/mL, Biolegend) in RPMI 1640 media supplemented with 10% fetal calf serum containing the following cytokines and antibodies: Recombinant Mouse IL-2 (carrier-free, Cat #: 575406, Biolegend) (20 ng/mL), anti-IFN-γ (10 µg/mL) and anti-IL-4 (10 µg/mL) for Th0; Recombinant Mouse IL-6 (carrier-free, Cat #: 575704, Biolegend) (20 ng/mL), Recombinant Human TGFβ1 (carrier-free, Cat #: 580702, Biolegend) (1 ng/mL), and Recombinant Mouse IL-23 (carrier-free, Cat #: 589002, Biolegend) (5 ng/mL) for Th17; TGF-β1 (1 ng/mL) for Treg cells. The Th0, Th17, and Treg cells were then rinsed with phosphate-buffered saline (PBS) and re-cultured in serum-free media for another 24 hours, and the supernatant ($CD4^+$ T cell-secreted factors, CM) was collected and used in subsequent experiments. The cells were snap-frozen in liquid nitrogen and stored at -80°C for subsequent experiments. See detailed antibody information in Supplementary Table 2.

Cell lines and cell culture

Human prostate cancer (PCa) cell lines (LNCaP, DU-145, and PC-3) and mouse PCa cell lines (MyC-CaP/CR) were purchased from the American Type Culture Collection (ATCC, Rockville, MD) and were authentic and free of mycoplasma [4]. LNCaP, DU-145, and PC-3 were grown in RPMI 1640 supplemented with 10% fetal bovine serum (FBS). MyC-CaP/CR cells were grown in DMEM supplemented with 10% FBS. These cells were exposed to CM for 48 hours, and then western blotting, immunocytochemistry (ICC), or immuno-fluorescence (IF) staining was performed for P-IκBα or NF-kB-p65.

Flow cytometry analysis

Splenocytes were isolated from *Batf*+ and *Batf*- mice as previously described [4]. The polarized Th17 and Treg cells from naïve $CD4^+$ T cells were rinsed with PBS re-cultured in RPMI 1640 medium containing 2 mM glutamine and 10% FBS in the presence of 50 ng/mL phorbol 12-myristate 13-acetate (PMA, Cat #: P1585, Sigma) and 1 mM ionomycin (Cat #: I0634, Sigma) for 6 hours. 1 mg/mL brefeldin A was added for the last 3 hours of culture (all purchased from Sigma Aldrich). After that, cells were stained for surface markers (anti-CD4, Code CL012AP, Cedarlane Labs). Intracellular cytokine staining (anti-IL-10, Cat No: 510504, BioLegend) was performed as described previously [4]. Approximately 100,000 cells were analyzed and only $CD4^+$ T cells were gated using a BD FACSymphonyTM A3 Cell Analyzer. See detailed antibody information in Supplementary Table 2.

Western blot analysis and qRT-PCR

Prostates were pulverized for protein or RNA extracts. Mouse splenocytes were treated with or without 5 ng/ml recombinant mouse IL-23 (carrier-free, Cat #: 589002, Biolegend) in

RPMI 1640 medium for 2 hours. Then, the cells were collected, and RNA was extracted for quantitative reverse transcription-PCR (qRT-PCR). $CD4^+$ cells isolated from the spleen were snap-frozen for protein or RNA extracts. Western blot analysis of Batf and related protein expression was performed as described [4]. mRNA level was analyzed by qRT-PCR as described [4]. Briefly, RNA was isolated using a RNeasy Plus Mini Kit (74136, Qiagen) and Direct-zolTM RNA MiniPrep Plus Kit (Cat. No: R2072) and cDNA synthesis with a PrimeScript™ Reagent Kit (Cat No: RR037A, TaKaRa. The resulting cDNA was used as a template for qRT-PCR performed with iTaq™ Universal SYBR®Green Supermix (Cat No: 172-5122, Bio-Rad) and a Thermal Cycler CFX Opus 96 Real-Time PCR System (Bio-Rad). PCR primer sequences used are listed in Supplementary Table 1.

Chromatin immunoprecipitation (ChIP) Assay

Mouse splenocytes were treated with or without 5 ng/ml IL-23 (Recombinant Mouse IL-23 (carrier-free), Cat #: 589002, Biolegend) in RPMI 1640 medium for 2 hours. Following the manufacturer's instructions, a ChIP assay was performed using an EZ-Magna ChIP™ G chromatin immunoprecipitation kit (catalog # 17-409, Millipore). Briefly, the cells were cross-linked with 1% formaldehyde. After stopping the cross-linking reaction with 10X glycine solution, the cells were lysed, and the lysates were sonicated to shear DNAs into 200- to 1000-bp fragments. Fifty microliters of chromatin extract from each sample were added to 450 ml dilution buffer. Five microliters of the mixture were aliquoted as "input"; the rest was mixed with 2 μg anti-BATF antibody and 20 μl protein G magnetic beads for overnight incubation with rotation at 4°C. The protein G magnetic beads were washed four times on the Magna GrIP rack (Millipore). Then, the beads were digested with proteinase K in 100 μl ChIP elution buffer at 60°C for two hours. Finally,

quantitative PCR (qPCR) was performed using the purified DNA. Results were normalized to DNA inputs using the formula ΔCt (cycle threshold) = Ct of DNA from immunoprecipitated (IP) $2^{-\text{Ct of DNA input}}$. The ΔCt of IgG IP was used as the baseline; therefore, ΔΔCt was calculated using the formula ΔΔCt = ΔCt of anti-BATF IP $2^{\Delta\text{Ct of IgG IP}}$ - ΔCt of anti-IgG IP $2^{\Delta\text{Ct of IgG IP}}$. MyC-CaP/CR cells were cultured in DMEM and transfected with 4μg pcDNA3.1-mBATF (Plasmid #34575, Addgene) for 48 hours. A ChIP assay was performed in the same way as above mentioned.

Enzyme-Linked Immunosorbent Assay (ELISA)

Mouse IL-17A and IL-23R in mouse serum were determined using ELISA kits (Mouse IL-17 DuoSet ELISA, Cat #: DY421-05; Mouse IL-23R DuoSet ELISA, Cat #: DY1686-05, R&D Systems, Minneapolis, MN) according to the manufacturer’s instructions. Values of all samples were expressed as means ± 95% confidence interval (CI) of triplicate measurements.

IL-23p19 antibody treatment

Eight 9-week-old male *Pten*-deficient mice were randomly distributed into the treatment group (n=4) and control group (n=4). The *Pten*-deficient mice were injected intravenously (i.v.) through the tail vein once a week with 2 mg/kg [mouse body weight (BW)] with rat anti-mouse IL-23p19 monoclonal antibody (treatment group) or control IgG (control group) for four weeks. The procedure is to prepare animals before injection, weigh each animal, calculate the injection volume according to the body weight and antibody concentration, and warm the animal for 5-10 minutes to dilate the veins. Then, lightly anesthetize the animal, insert the needle (small gauge, 27-30), bevel up into the vein towards the direction of the head, and slowly inject. Remove the needle and apply gentle compression until the bleeding has stopped. Monitor the animal during

recovery, return animals to their cage, and observe to ensure that bleeding has not resumed. IL-23p19 Monoclonal Antibody (G23-8), Functional Grade, eBioscience™, Cat # 16-7232-81 (50 μg), Cat # 16-7232-85 (500 μg); control IgG: Cat # 16-4301-85, eBioscience Inc. San Diego, CA. The antibodies were dissolved in saline. The injection time points were 9-, 10-, 11-, and 12-week-old. All animals were euthanized for necropsy at 16 weeks of age. See detailed antibody information in Supplementary Table 2.

Statistics

Statistical analysis was performed using R Software (R Foundation for Statistical Computing, Vienna, Austria). Comparisons of the GU-bloc weights were analyzed with Student's *t*-test and analysis-of-variance (ANOVA). Student's *t*-test was used to analyze the remaining data. All *in vitro* experiments were repeated at least three times. Data are presented if not indicated elsewhere as mean ± 95% CI. Statistical significance is indicated in all figures by the following annotations: **P* <0.05; ***P* <0.01; ****P* <0.001; *****P* <0.0001; ns = not significant.

References:

1. Wang S, Gao J, Lei Q, Rozengurt N, Pritchard C, Jiao J, et al. **Prostate-specific deletion of the murine Pten tumor suppressor gene leads to metastatic prostate cancer**. *Cancer Cell* 2003; 4(3):209-221.

2. Wu X, Wu J, Huang J, Powell WC, Zhang J, Matusik RJ, et al. **Generation of a prostate epithelial cell-specific Cre transgenic mouse model for tissue-specific gene ablation**. *Mech Dev* 2001; 101(1-2):61-69.

3. Schraml BU, Hildner K, Ise W, Lee WL, Smith WA, Solomon B, et al. **The AP-1 transcription factor Batf controls T(H)17 differentiation**. *Nature* 2009; 460(7253):405-409.

4. Liu S, Liu F, Zhang B, Yan P, Rowan BG, Abdel-Mageed AB, et al. **$CD4^+$ T helper 17 cell response of aged mice promotes prostate cancer cell migration and invasion**. *Prostate* 2020; 80(10):764-776.

5. Shappell SB, Thomas GV, Roberts RL, Herbert R, Ittmann MM, Rubin MA, et al. **Prostate pathology of genetically engineered mice: definitions and classification. The consensus report from the Bar Harbor meeting of the Mouse Models of Human Cancer Consortium Prostate Pathology Committee**. *Cancer Res* 2004; 64(6):2270-2305.

6. Tian H, Huo Y, Zhang J, Ding S, Wang Z, Li H, et al. **Disruption of ubiquitin specific protease 26 gene causes male subfertility associated with spermatogenesis defects in mice†**. *Biol Reprod* 2019; 100(4):1118-1128.

7. Liu S, Zhang B, Rowan BG, Jazwinski SM, Abdel-Mageed AB, Steele C, et al. **A Novel Controlled PTEN-Knockout Mouse Model for Prostate Cancer Study**. *Front Mol Biosci* 2021; 8:696537.

Supplementary Table 1. PCR primer sequences[a]

Species	Gene	Primer	Nucleotide sequence (5' to 3')
Mouse qPCR	*Batf*	sense	CACAGAAAGCCGACACCCTTCA
		antisense	GCTGCTCAGCACTGATGTGAAG
	Il23	sense	AATAATGTGCCCCGTATCCAGT
		antisense	GCTCCCCTTTGAAGATGTCAG
	Il23r	sense	GAGGACATCCTGCTTCAGGTAAT
		antisense	AGCCACTTTGGGATCATCAGTA
	Il17a	sense	AGGCAGCAGCGATCATCC
		antisense	GTGGAACGGTTGAGGTAGTC
	Il17f	sense	CCCAGGAAGACATACTTAGAAGAAA
		antisense	CAACAGTAGCAAAGACTTGACCA
	Il10	sense	GGGAGAACCTGAAGACCCTCA
		antisense	TGCTCTTGTTTTCACAGGGAAG
	Rorc (Rorγt)	sense	AGTCGTCCTAGTCAGAATG
		antisense	ATGTTCCACTCTCCTCTTC
	Foxp3	sense	ACCATTGGTTTACTCGCATGT
		antisense	TCCACTCGCACAAAGCACTT
	Gapdh	sense	TGGCCTTCCGTGTTCCTAC
		antisense	GAGTTGCTGTTGAAGTCGCA
Mouse ChIP qPCR	*Il23r Primer pair 1*	sense	GGAGGAACTGAGAGGTCTAAATC
		antisense	GCACATAGGAGACAGAGGTAAG
	Il23r Primer pair 2	sense	TGCTTACCTCTGTCTCCTATGT
		antisense	TGGCTGTGCTGTCTTCTTTC

[a]All primers were ordered from Eurofins Genomics, Louisville, KY.

Supplementary Table 2. Antibodies used in this study

Antibody	Primary validation	Catalog #	Uses
**IFN-γ**	Biolegend	505827	Th differentiation
**IL-2**	Biolegend	503702	Th differentiation
**IL-4**	Biolegend	504122	Th differentiation
**Arginase I (V-20)**	Santa Cruz Biotechnology, Inc.	sc-18354	IHC
**BATF antibody produced in rabbit**	Millipore, Temecula, CA	SAB4500122	IHC/WB/IP
**Bcl-xL (54H6) Rabbit mAb**	Cell Signaling Technology	2764s	IHC
**Cleaved caspase-3**	Cell Signaling Technology	9664S	IHC
**Cleaved Caspase-7 (Asp198) (D6H1) Rabbit mAb**	Cell Signaling Technology	8438S	IHC
**Cleaved Caspase-9 (Asp330) (E5Z7N) Rabbit mAb**	Cell Signaling Technology	52873T	IHC
**CD25 - PE-Cyanine7**	Invitrogen	25-0251-82	FACS
**CD28 Monoclonal Antibody (37.51), Functional Grade, eBioscience™**	Invitrogen	16-0281-82	Th differentiation
**CD3e Monoclonal Antibody (145-2C11), Functional Grade, eBioscience™**	Invitrogen	16-0031-85	Th differentiation
**CD3e Monoclonal Antibody (145-2C11), PE, eBioscience™**	Invitrogen	12-0031-82	FACS
**CD31**	Abcam, PLC., Cambridge, MA	Ab28364	IHC
**Anti-Cytokeratin 19/KRT19 Antibody Picoband™**	Boster Biological Technology	PB9715	IHC
**Anti-Cytokeratin 8/KRT8 Antibody Picoband™**	Boster Biological Technology	A01421	IHC
**Anti-Mouse CD4 Purified (Clone YTS 191.1) (rat IgG2b)**	Cedarlane Labs	CL012AP	IHC
**Brilliant Violet 421™ anti-mouse CD4 Antibody**	Biolegend	116023	FACS
**Purified anti-mouse CD8a Antibody**	Biolegend	100702	IHC
**FITC anti-mouse CD8a Antibody**	Biolegend	100706	FACS
**Cleaved-PARP (Asp214) (D64E10)**	Cell Signaling Technology	5625	IHC
**FOXP3 Monoclonal Antibody (FJK-16s), eBioscience™**	Invitrogen	14-5773-82	IHC

**FoxP3-APC**	eBioscience	17-5773-82	FACS
**Rat IgG1 kappa Isotype Control (eBRG1), Functional Grade, eBioscience™**	Invitrogen (eBioscience)	16-4301-85	Treatment
**Rat IgG2a kappa Isotype Control (eBR2a), APC, eBioscience™**	eBioscience	17-4321-81	FACS
**Brilliant Violet 421™ Mouse IgG2b, κ Isotype Ctrl Antibody**	Biolegend	400342	FACS
**Rat IgG2a kappa Isotype Control (eBR2a), FITC, eBioscience™**	eBioscience	11-4321-41	FACS
**PE Rat IgG2a, κ Isotype Ctrl Antibody**	Biolegend	400508	FACS
**Rat IgG1 kappa Isotype Control (eBRG1), PE-Cyanine7, eBioscience™**	eBioscience	25-4301-81	FACS
**IκBα (L35A5) Mouse mAb**	Cell Signaling Technology	4814S	WB
**Purified anti-human IL-10 Antibody**	BioLegend	501504	IHC
**IL-17 (E19)**	Santa Cruz Biotechnology, Inc.	SC-6077	IHC
**IL-23p19 Monoclonal Antibody (G23-8), Functional Grade, eBioscience™**	Invitrogen (eBioscience)	16-7232-85	Treatment
**IL-23R**	Millipore Sigma.	06-1331	IHC
**Ki-67**	Millipore, Temecula, CA	AB9260	IHC
**Anti-Laminin antibody produced in rabbit**	Sigmaaldrich	L9393	IHC
**NF-κB/p65 (C-20)**	Santa Cruz Biotechnology, Inc.	sc-372	IHC
**Phospho-IκBα (Ser32) (14D4) Rabbit mAb**	Cell Signaling Technology	2859S	WB
**CD3 Monoclonal Antibody (eBioG4.18 (G4.18)), PE, eBioscience™**	Invitrogen	12-0030-82	FACS
**P-S6 (Ser240/244) (D68F8) XP**	Cell Signaling Technology	5364S	IHC
**Sox-9 Antibody (H-90)**	Santa Cruz Biotechnology, Inc.	sc-20095	IHC
**BD Horizon™ PE-CF594 Mouse Anti-Mouse RORγt**	BD Bioscience	562684	FACS

**ROR gamma (t) Monoclonal Antibody (AFKJS-9), eBioscience™**	Invitrogen	14-6988-82	IHC
**TP63 Recombinant Rabbit Monoclonal Antibody (10H7L17)**	ThermoFisher Scientific	703809	IHC

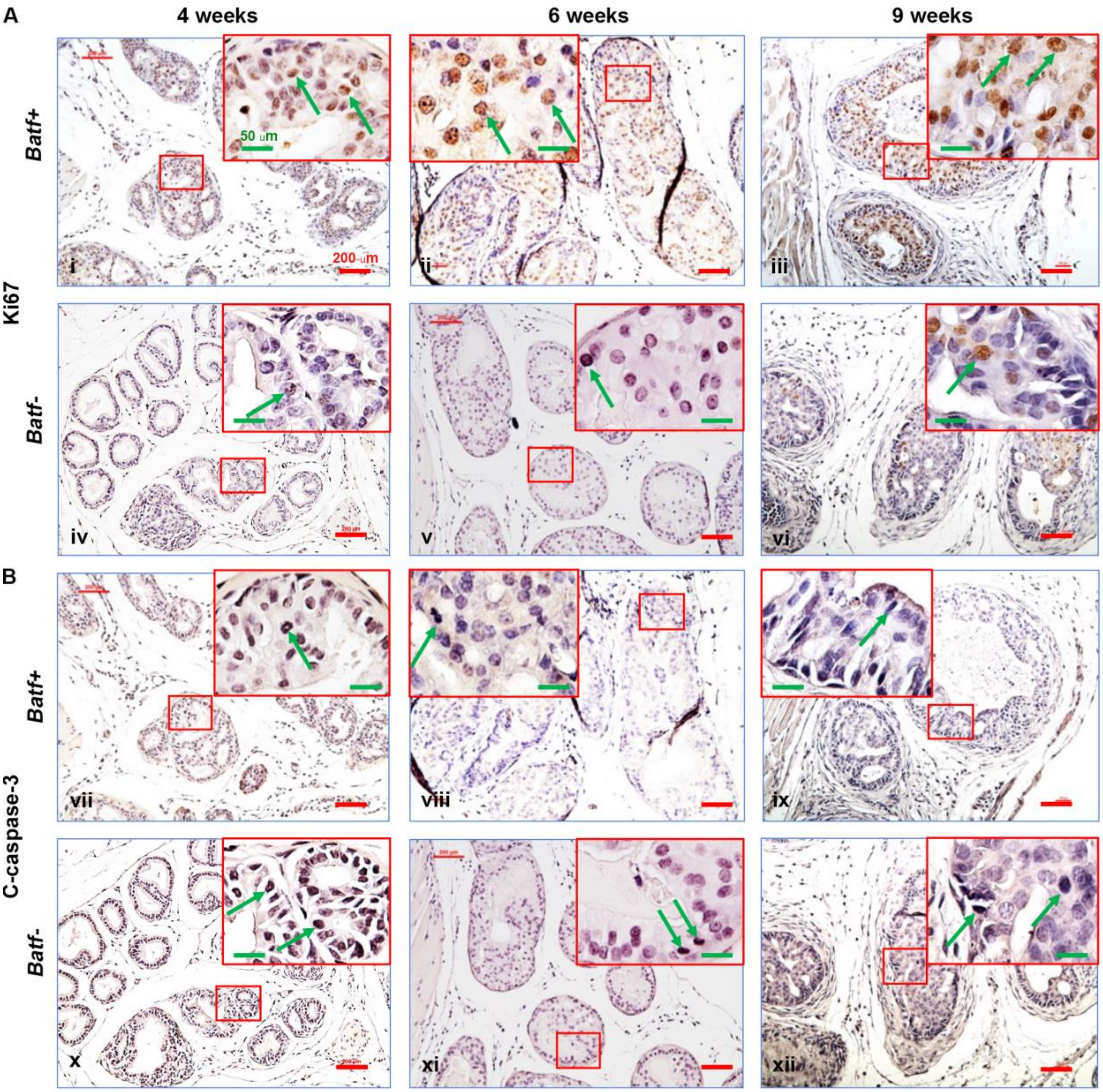

Supplementary Figure 1. *Batf* knockout decreased cellular proliferation, increased apoptosis, and reduced angiogenesis in prostate tissues. **A)** Representative IHC staining for Ki-67 in 4, 6, and 9 weeks of age *Batf*+ and *Batf*- mouse prostate tissues; arrows indicate the positive cells. Original magnification, x100 (scale bar, 200 μm), inserts x400 (scale bar, 50 μm). **B**) Representative IHC staining for C-caspase-3 in 4, 6, and 9 weeks of age *Batf*+ and *Batf*- mouse prostate tissues; arrows indicate the positive cells. Original magnification, x100 (scale bar, 200 μm), inserts, x400 (scale bar, 50 μm). Ai-Avi and Bvii-Bxii are consecutive sections for each genotype and age group.

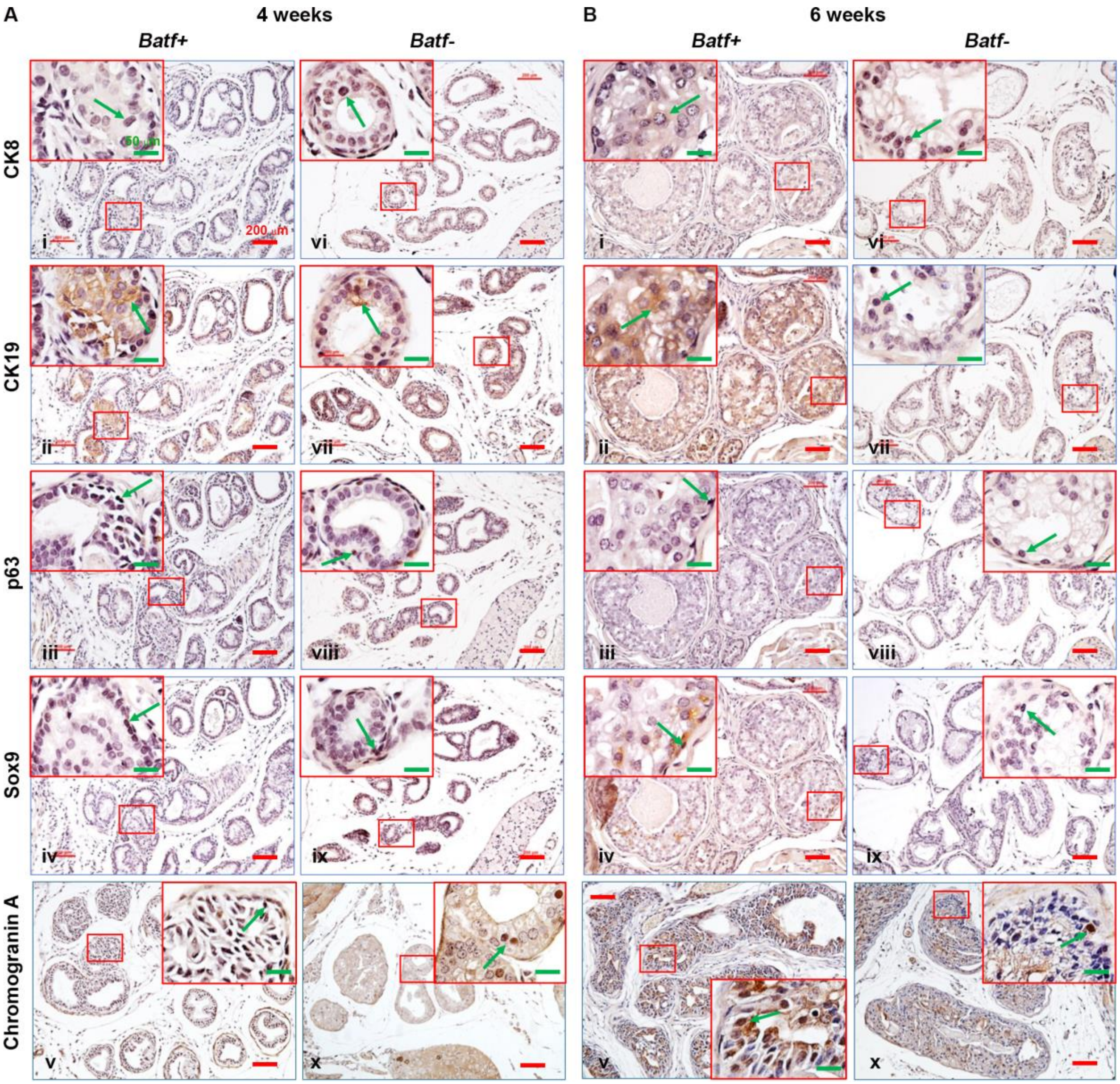

Supplementary Figure 2. *Batf* knockout decreased the expression of cytokeratin, stem cells, and neuroendocrine markers in the prostate gland epithelia at 4 and 6 weeks of age. **A)** Representative IHC staining for CK8, CK19, p63, SOX9, and Chromogranin A (**Av** and **Ax**) in 4 weeks of age *Batf*+ and *Batf*- mouse prostate tissues. **B)** Representative IHC staining for CK8, CK19, p63, SOX9, and Chromogranin A (**Bv** and **Bx**) in 6 weeks of age *Batf*+ and *Batf*- mouse prostate tissues. Arrows indicate the positive cells. Original magnification, x100 (scale bar, 200 μm), inserts, x400

(scale bar, 50 μm). **Ai-Aiv**, **Avi-Aix**, **Bi-Biv**, and **Bvi-Bix** are consecutive sections for each genotype and age group.

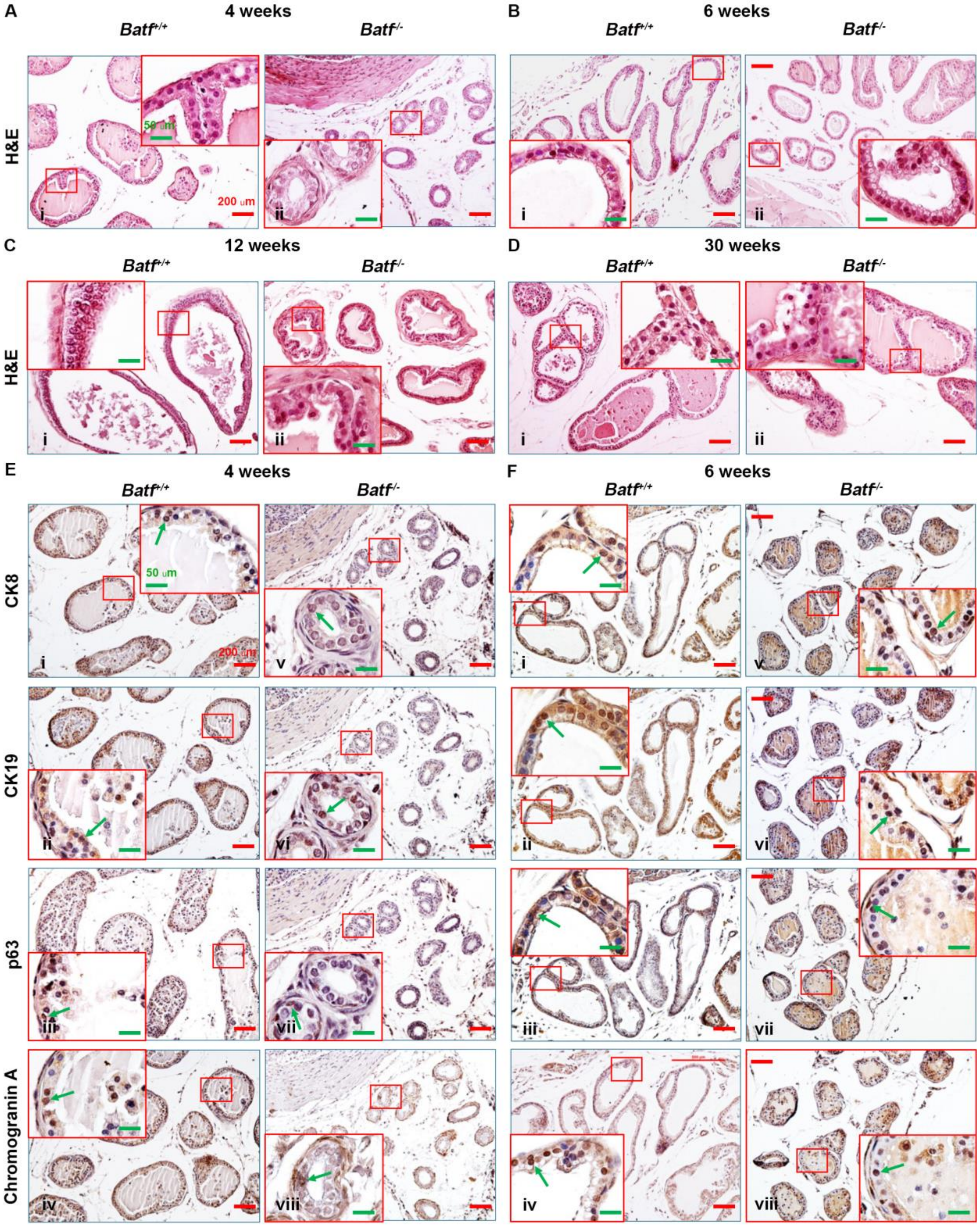

Supplementary Figure 3. *Batf* knockout did change the morphology of prostate tissue. Still, no cancer development, the expression of cytokeratin, basal cell, and neuroendocrine markers reduced

in the prostate gland epithelia compared to Batf expression mice at 4 and 6 weeks of age with *Pten* WT. **A-D)** Representative H&E-stained dorsal prostate lobes at 4, 6, 12, and 30 weeks of age. Original magnification, x100 (scale bar, 200 μm), inserts, x400 (scale bar, 50 μm). **E)** Representative IHC staining for CK8, CK19, p63, and Chromogranin A in 4 weeks of age *Batf*$^{+/+}$ and *Batf*$^{-/-}$ mouse prostate tissues. **F)** Representative IHC staining for CK8, CK19, p63, and Chromogranin A in 6 weeks of age *Batf*$^{+/+}$ and *Batf*$^{-/-}$ mouse prostate tissues. Arrows indicate the positive cells. Original magnification, x100 (scale bar, 200 μm), inserts, x400 (scale bar, 50 μm). **Ei-Eiv**, **Ev-Eviii**, **Fi-Fiv**, and **Fv-Fviii** are consecutive sections for each genotype and age group.

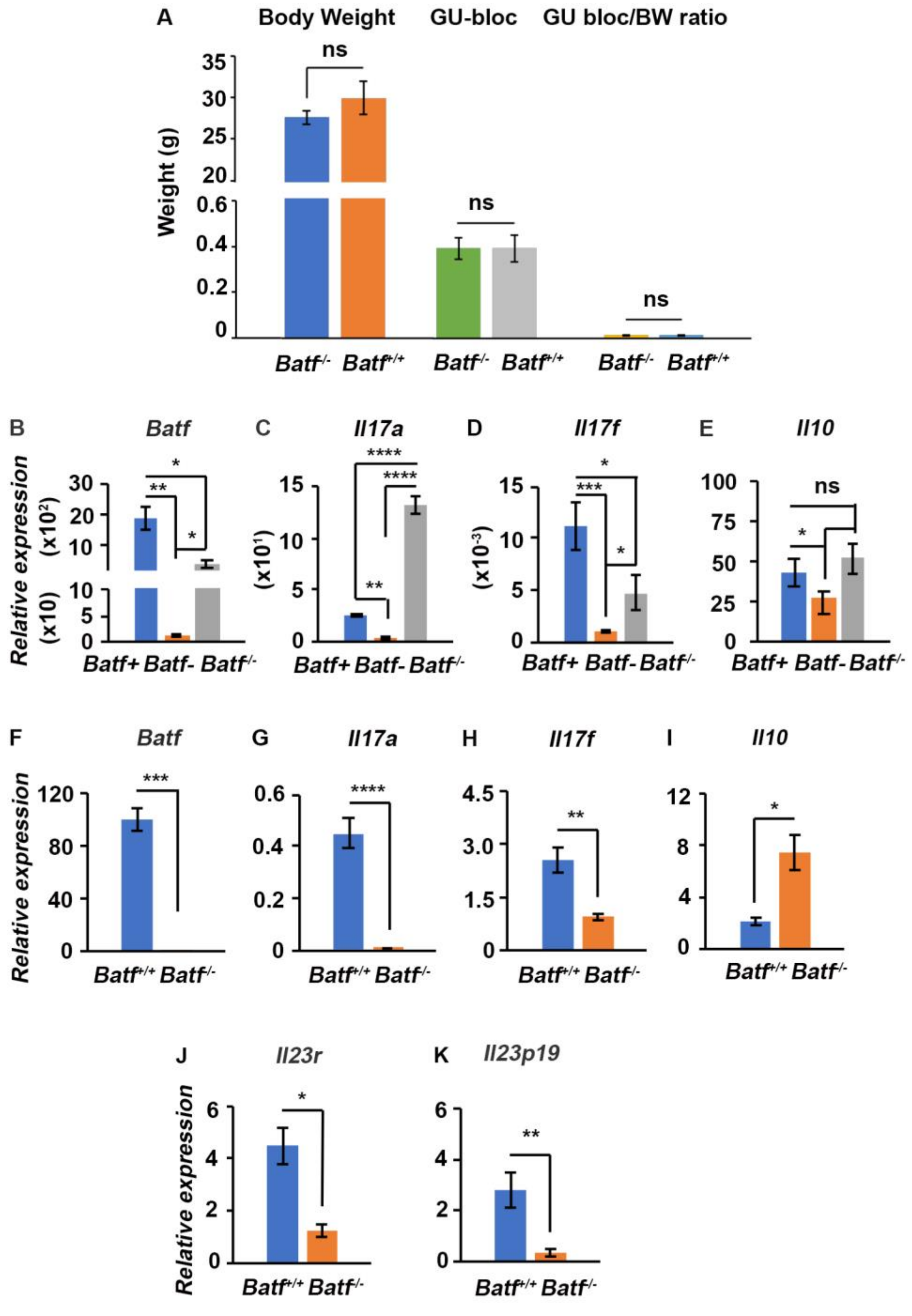

Supplementary Figure 4. *Batf* knockout did not change the *Pten* WT mouse body weight, GU bloc weight, and GU bloc/BW ratio but decreased the expression of Th17-related cytokines, *Il23r*, and *Il23p19*, increased the expression of *Il10* in *Pten* WT mouse prostate tissue. **A)** Body weight, GU bloc weight, and GU-bloc/BW ratio of 20-30-week-old *Batf*$^{-/-}$ and *Batf*$^{+/+}$ mice. n=9 (*Batf*$^{-}$

$^{/-}$ mice), n=6 (*Batf*$^{+/+}$ mice), ns, insignificant. **B-E)** qPCR results for *Batf, Il17a, Il17f, and Il10 in Batf+, Batf-, and Batf*$^{-/-}$ mice prostate tissue. **P* < 0.05, ***P* < 0.01, ****P* < 0.001, *****P* < 0.0001, ns, not significant (this is the same result as in Figure 4A using different ways to show the same results). **F-K)** qPCR results for *Batf, Il17a, Il17f, Il10, Il23r,* and *Il23p19* in *Batf*$^{+/+}$ versus *Batf*$^{-/-}$ (all *Pten* WT) mouse prostate tissue. **P* < 0.05, ***P* < 0.01, ****P* < 0.001, *****P* < 0.0001.

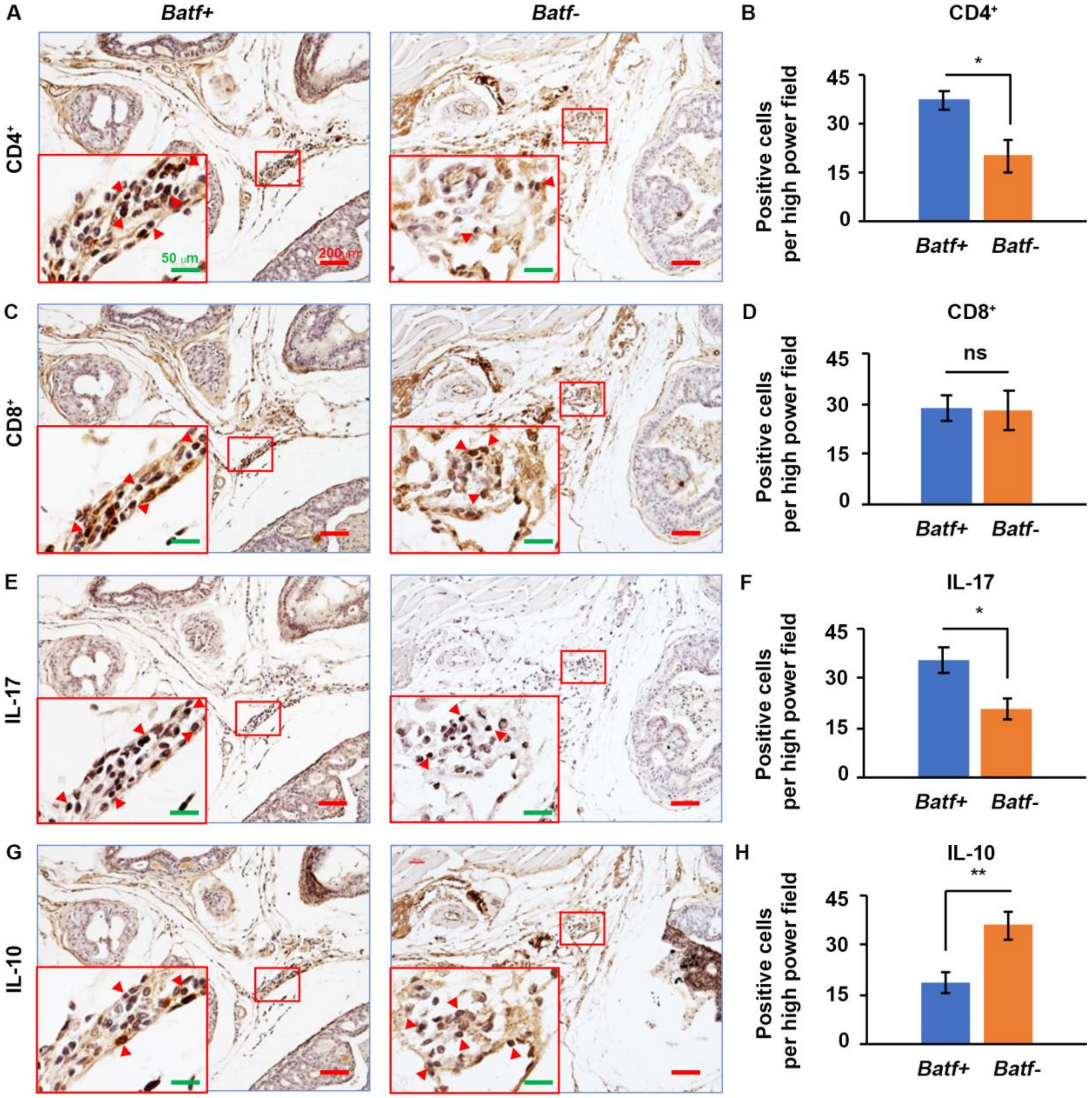

Supplementary Figure 5. *Batf* knockout decreased immune cells in mouse prostate stroma. **A, C, E, G)** Representative IHC staining for CD4, CD8, IL-17 and IL-10-expressing cells in the prostate stroma of 30-week-old *Pten*-null mice. **A, C, E, and G** are consecutive sections from *Batf+* or *Batf-* mouse prostate tissue. Original magnification: x100 (scale bar, 200 μm), inserts, x400 (scale bar, 50 μm). **B, D, F, H)** Number of positive cells per high power field. **P* < 0.05, ***P* < 0.01, ns, insignificant.

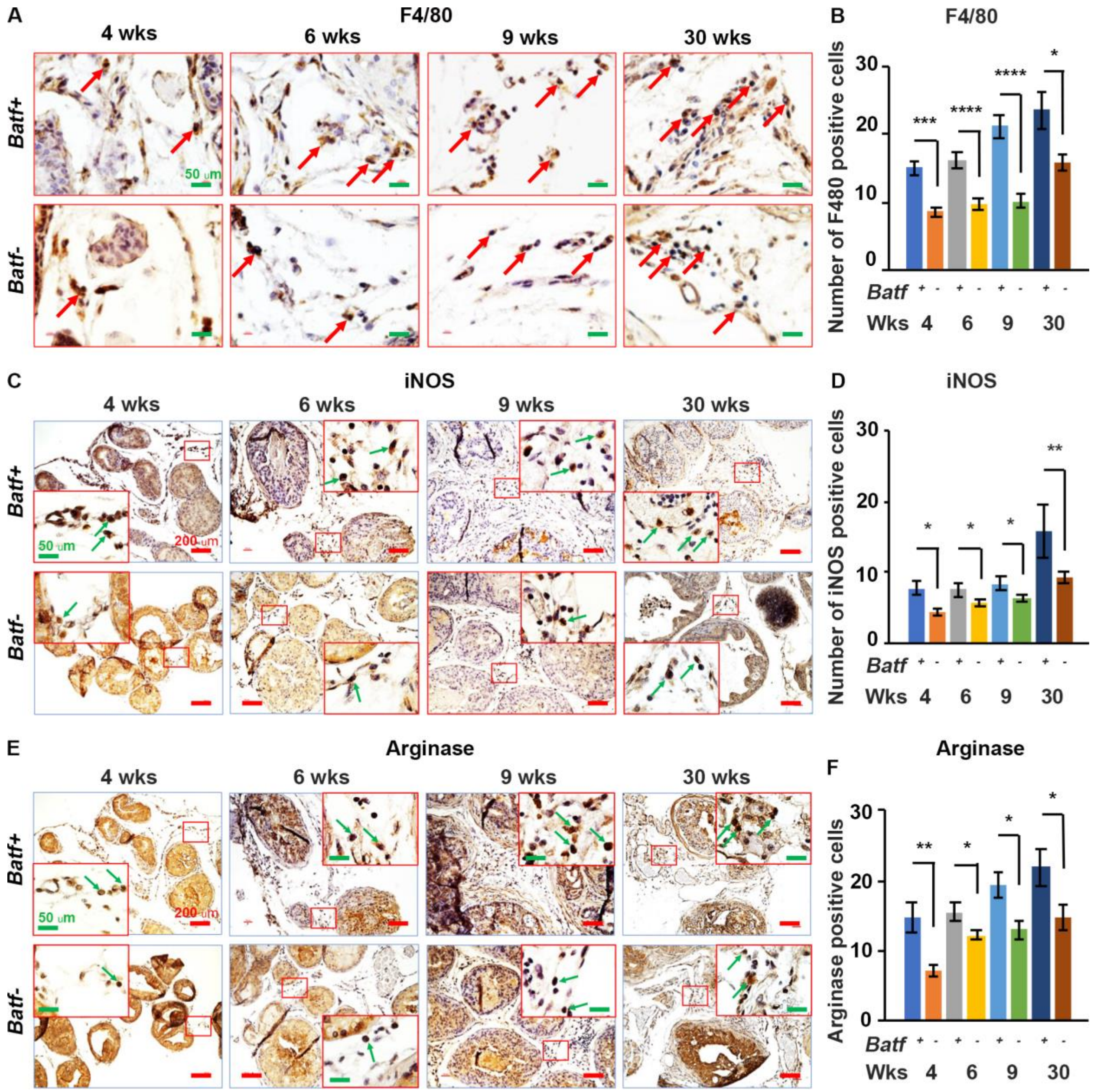

Supplementary Figure 6. *Batf* knockout decreased macrophages in mouse prostate stroma. **A, C, E)** Representative IHC staining for F4/80 (macrophage marker), iNOS (M1 macrophage marker), and Arginase (M2 macrophage marker) in the prostate stroma of mice at 4, 6, 9, and 30 weeks of age. The arrows indicate the positive cells. Original magnification, **A**, x400 (scale bar, 50 μm), **C & E**, x100 (scale bar, 200 μm), inserts, x400 (scale bar, 50 μm). **B, D, F)** Number of positive cells

for F4/80, iNOS, and Arginase per high power field. $*P < 0.05$, $**P < 0.01$, $***P < 0.001$, $****P < 0.0001$.

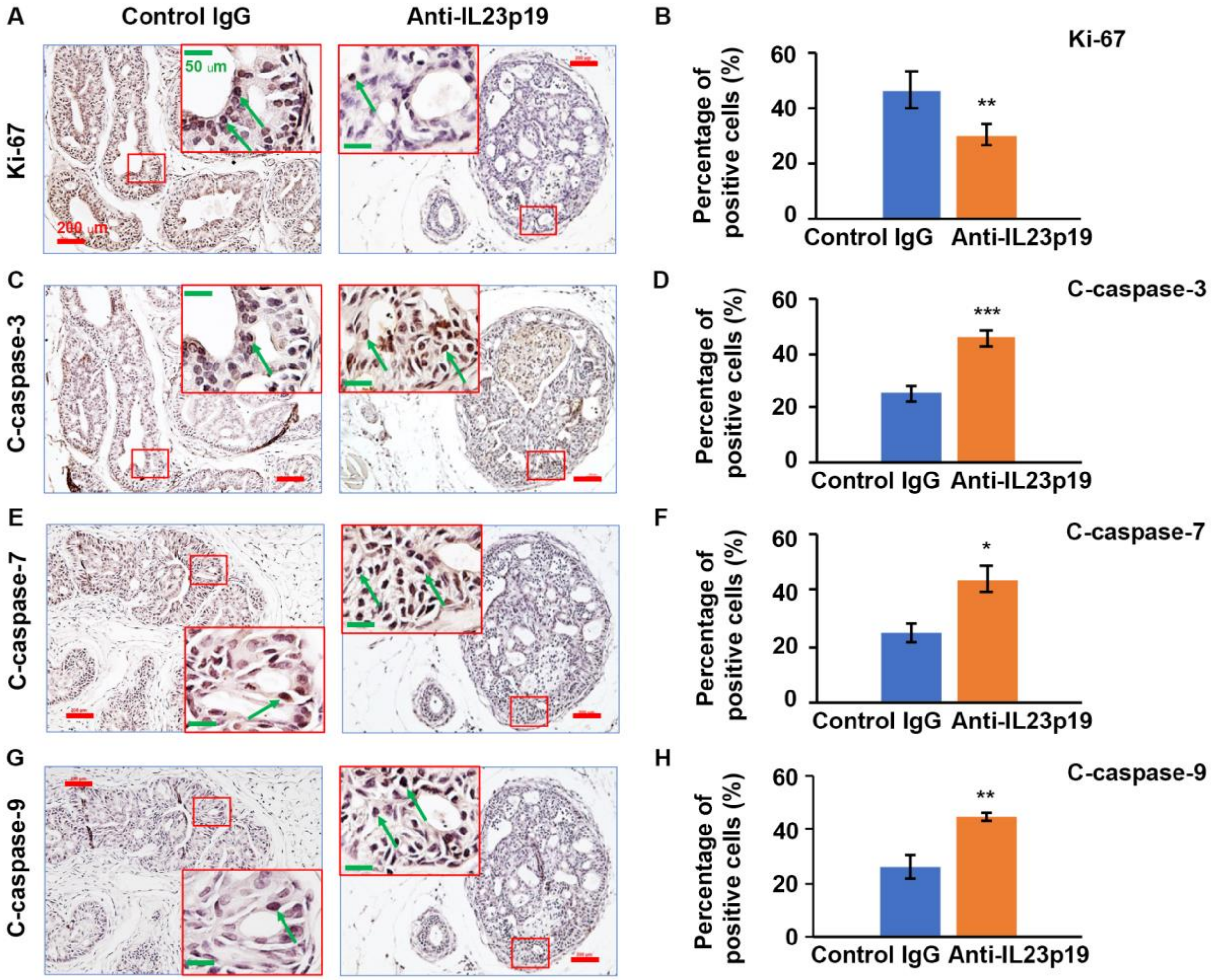

Supplementary Figure 7. Anti-IL23p19 antibody treatment decreased cell proliferation and increased apoptosis in the prostate epithelia in *Pten*-null mice. **A, C, E, G)** Representative IHC staining for Ki-67, Cleaved caspase-3 (C-caspase-3), C-caspase-7, and C-caspase-9 in the prostate epithelia of *Pten*-null mice treated with anti-IL23p19 or control IgG. All the sections are consecutive; the arrows indicate the positive cells; original magnification, x100 (scale bar, 200 μm), inserts, x400 (scale bar, 50 μm). **B, D, F, H)** Percentage of positive cells per high power field. *$P < 0.05$, **$P < 0.01$, ***$P < 0.001$.

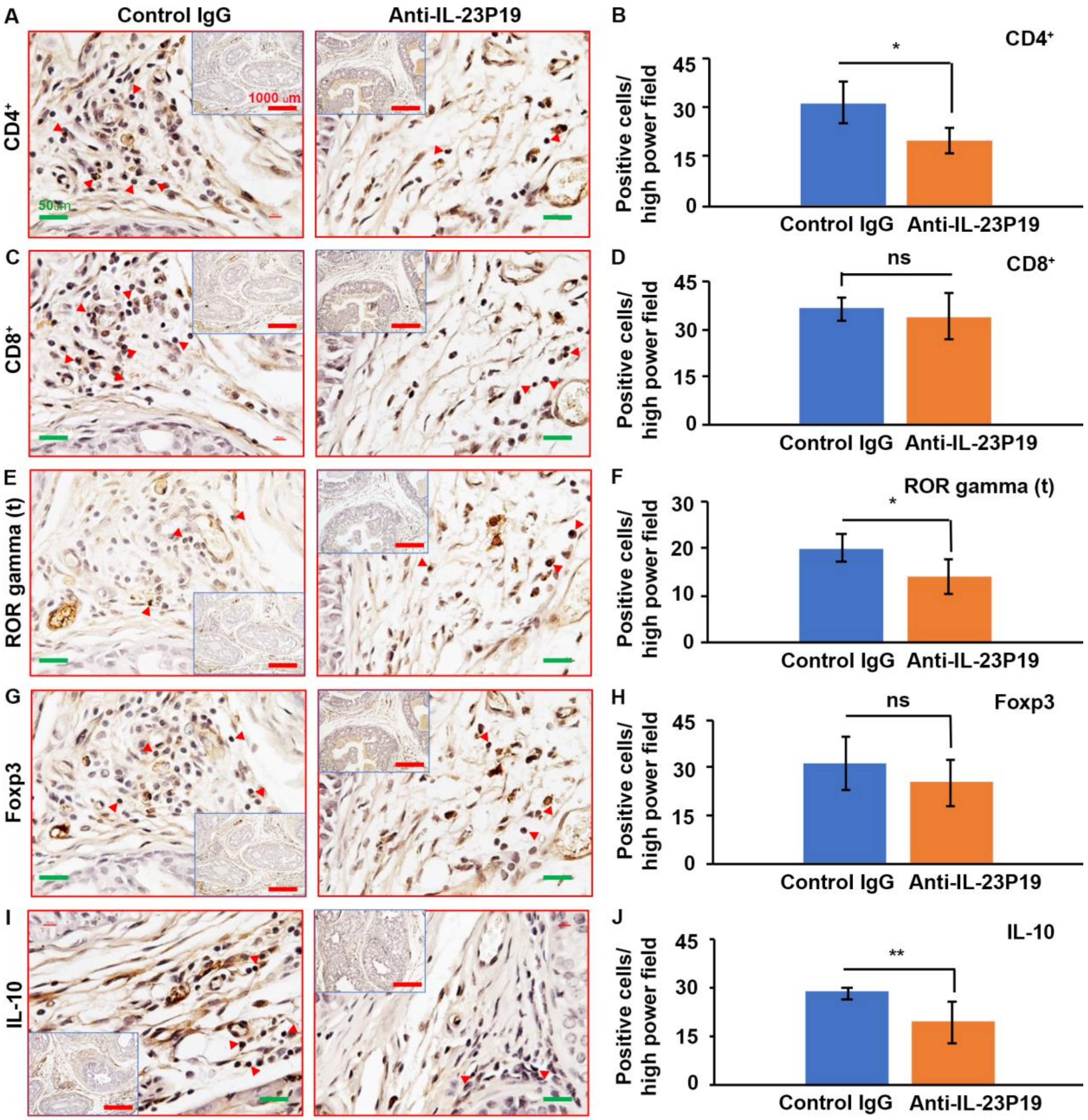

Supplementary Figure 8. Anti-IL-23p19 antibody treatment decreased immune cells in the prostate stroma in *Pten*-null mice. **A, C, E, G, I)** Representative IHC staining for CD4, CD8, ROR gamma (t) (RORγt), Foxp3, and IL-10-expressing cells in the prostate stroma of anti-IL-23p19 or control IgG-treated *Pten*-null mice. **A, C, E, G** are consecutive sections from the anti-IL-23p19 or control IgG treatment group. The sections in **I** are consecutive sections from **Figure 8, A** and **C.** The arrowheads indicate the positive cells. Original magnification: x400 (scale bar, 50 μm),

inserts, x100 (scale bar, 1000 μm). **B, D, F, H, J)** Number of positive cells per high power field. *$P < 0.05$, **$P < 0.01$, ns, insignificant.
